# *In vitro* and *in silico* studies of the interaction between glucocorticoid drug mometasone furoate and model lung surfactant monolayer†

**DOI:** 10.1039/d5ra00004a

**Published:** 2025-02-26

**Authors:** Md. Zohurul Islam, Martyna Krajewska, Krystyna Prochaska, Suvash C. Saha

**Affiliations:** a Department of Mathematics, Faculty of Science, Jashore University of Science and Technology Jashore-7408 Bangladesh mz.islam@just.edu.bd; b High Performance Computing (HPC) Laboratory, Department of Mathematics, Jashore University of Science and Technology Jashore-7408 Bangladesh; c Institute of Chemical Technology and Engineering, Poznan University of Technology Berdychowo 4 60-965 Poznań Poland; d School of Mechanical and Mechatronic Engineering, University of Technology Sydney 15 Broadway Ultimo NSW 2007 Australia

## Abstract

In an effort to treat preterm neonates who are already suffering or are at high risk for chronic pulmonary illness, the comprehensive investigation has recently been focused on the intratracheal administration of corticosteroid drugs employing an innate lung surfactant as a drug carrier. A novel approach to utilize exogenous surfactant preparation as a drug delivery vehicle for corticosteroids, which are the inflammation-reducing agents for lung diseases has not been comprehensively investigated. The direct corticosteroid drugs administered through pulmonary surfactants would impair their surface activity and exacerbate normal breathing cycles. This study was conducted to characterize the physiological interaction between frequently used inhaled corticosteroid, mometasone furoate, and relevant composition of lung surfactants by using the *in vitro* and *in silico* methods. The major objective of this work is to elucidate the effects of glucocorticoids on the structural and dynamical characteristics of the lung surfactant as well as the effects of the drug on the ability of the surfactant monolayer to reduce surface tension during mechanical breathing. Our results from the Langmuir experiment and atomic force microscopy imply that mometasone furoate concentrations less than 4.18% w/w might not strongly influence the physicochemical characteristics of the surfactant molecules representing the feasible concentration for pulmonary drug delivery. Beyond this range, mometasone furoate concentrations cause intensified film fluidization that leads the surfactant film to collapse at lower surface pressure, which is also verified by the *in silico* study. The failure of the drug to permeate into the lipid bilayer is most likely what causes this collapse. On the other hand, for inhalation breathing, the monolayer forms pores induced by the high drug concentrations. Our investigation also reveals that mometasone furoate exhibits different spreading behaviors because of their affinities to the surfactant molecules. This work may have implications for the use of inhaled steroids in the treatment of asthma in addition to its translational significance in the management of chronic lung disease.

## Abstract 2

The precise procedure for examining how the corticosteroid drug mometasone furoate interacts with a model lung surfactant monolayer.

## Graphical abstract

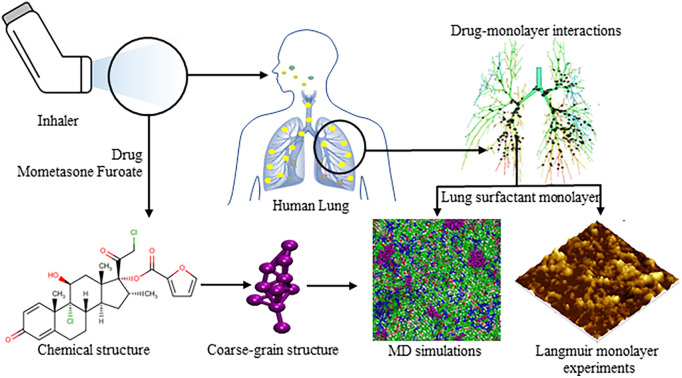

## 1 Introduction

Inhaled corticosteroids (ICS) persist as a potential anti-inflammatory therapy for chronic asthma.^1,2^ They can be found in the market with different generic names including Budesonide, Beclomethasone, Beclomethasone dipropionate, Flunisolide, Fluticasone propionate, Mometasone furoate (MF), and Ciclesonide.^3^ It has long been known that minor chemical alterations to the core corticosteroid molecule result in substantial variations in potency, which are typically gauged by their ability to bind to the glucocorticoid receptor.^4^ In order to understand how drugs influence the binding capacity with glucocorticoid receptors, it is crucial to investigate the drug–receptor or drug–membrane interaction mechanism. Such interaction includes essential functions for the pharmacokinetics and pharmacodynamics of the drug by controlling how quickly drugs spread and accumulate. The binding affinity for human serum albumin (HSA) is one of the crucial variables influencing the allocation of active concentrations of many corticosteroid medicines that are delivered to the lung alveolar surface.^5^ In contrast to oral and intravenous drug administration, inhalation is the recommended method for administering a corticosteroid because it allows the medicine to be delivered straight to the alveolar airways, and it reduces systemic side effects.^6^ Clinical investigations have demonstrated that inhaled corticosteroids considerably decrease airway hyperresponsiveness, successfully stop acute exacerbations, enhance lung function, and lessen the intensity of symptoms.^7^ In addition to their involvement in other physiological processes, corticosteroids also affect the release of inflammatory mediators in the respiratory pathways, including those produced by mast cells, eosinophils, lymphocytes, and macrophages.^8^ Freely circulating inhaled corticosteroids have the potential to bind with non-pulmonary glucocorticoid receptors and have negative consequences, such as reducing the activity of the hypothalamic–pituitary–adrenal (HPA) axis and impairing growth.^9^ It is possible to lessen the systemic adverse effects of inhaled corticosteroids by the extensive protein binding that helps to inhibit the side effects of the drug. To understand the drug–membrane interaction, an in-depth investigation needs to be conducted to observe the pharmacokinetics and pharmacodynamics mechanism of the corticosteroid drug. Pontremoli *et. al.*^5^ conducted a spectroscopic study to calculate the protein binding affinity of several inhaled drugs including betamethasone, flunisolide, prednisolone, and triamcinolone with HSA to see the pharmacokinetic behaviors of the corticosteroids.

Recently, much effort has been focused on the delivery of intratracheal corticosteroids utilizing a natural pulmonary surfactant for premature neonates with recurring pulmonary disease. Corticosteroids supplied as the anti-inflammatory drug and the surfactant preparation employed as the drug carrier, as well as their mixing ratios, have not been optimized yet. Pulmonary drug delivery has drawn more attention recently because it is a noninvasive technique that is capable of avoiding systemic side effects.^10^ The huge surface area (∼70–140 m^2^) of the alveoli in human lungs and the incredibly thin diffusive layer (<0.5 μm) in the peripheral lung alveoli are the significant elements contributing to the viability of respiratory drug administration.^11^ The lung airways are a good absorptive location for inhaled drugs due to these anatomical characteristics, making them a prospective track for the transport of medicines.^12^ Topical application to the lungs avoids first-pass metabolism and reduces the possibility of negative effects brought by high systemic dosage, in addition to facilitating drug transport more easily than intravenous injection.^13^ Because of these features, respiratory drug transportation has become a promising drug delivery approach for facilitating the management and treatment of pulmonary conditions including asthma and chronic pulmonary diseases. On top of the regional delivery, the respiratory tract can also be used for the complete administration of medicinal substances, particularly peptide and protein-based medicines.

Due to the harmful side effects of the systemic administration of corticosteroids, the frequent use of such drugs has been limited^14^ to treat local inflammation while minimizing harmful systemic consequences effectively.^15^ Clinical trials have been performed on two separate pulmonary delivery techniques. They are intratracheal installation of corticosteroids employing exogenous surfactant as a distribution agent, and very recently, inhalation administration of corticosteroid aerosols.^16,17^ Exogenous surfactants can be used as a drug transport agent into the alveoli, as found by *in silico*, *in vitro* and *in vivo* biomechanical, biophysical, or biochemical tests.^18,19^ The transportation of corticosteroid drugs together with the natural surfactant as the delivery vehicle in the targeted region shows multiple benefits. Firstly, a natural surfactant has excellent biocompatibility and biodegradability since it may either be taken up by the alveolar macrophages or expelled from the alveolar surface by endocytosis back into alveolar type II cells, where the natural lung surfactants are produced.^20,21^ Secondly, these hydrophobic corticosteroid drugs become more soluble in water by the combination of natural surfactant and drug molecules through a drug solubilization process.^22^ Thirdly, the spontaneous diffusion of natural surfactant relies on the differential surface tension at the alveolar interior layer of the airways, which occurs due to the Marangoni effect.^23,24^ This enables easy intratracheal delivery of corticosteroids along with natural surfactants to the distal lung.

Pulmonary surfactants are produced and excreted by pneumocyte type II cells and form an ultrafine layer at the air–water interface in the alveoli, which is referred to as the lung surfactant monolayer (LSM).^25,26^ In order to assist the mechanical work of breathing, LSM lowers surface tension by approaching 0 mN m^−1^ at the end of exhalation breathing and prevents alveolar collapse.^27,28^ In addition to protecting alveoli against various inhaled substances, LSM acts as the first line of defense against potentially inhaled microorganisms and/or particles reaching the respiratory airways. The lung surfactant constituents and the arrangement of the surfactant molecules at the air–liquid interface of air sacs are critical to various functions, including the modulation of alveolar space and the pulmonary surfactant deformability.^29^ Any imbalance of lung surfactant components (phospholipids, cholesterol, and proteins) reduces the effectiveness of lung functioning. Phospholipids comprise around 85% of the weight of mammalian lung surfactant components, making them the most prevalent substance. The zwitterionic phosphatidylcholine (PC) lipids are the highest prevalent species of phospholipids, constituting approximately 70% of the total weight. The di-saturated phospholipid 1,2-dipalmitoyl-*sn-glycero*-3-phosphatidylcholine (DPPC) makes up more than half of the total PC lipids, whereas the residual species of lipids are mono- or di-unsaturated phospholipids such as 1,2-palmitoyl-2-oleoyl-*sn-glycero*-3-phosphatidylcholine (POPC).^30,31^ The following key phospholipids are negatively charged phosphatidylglycerol (PG) lipids, such as 1-palmitoyl-2-oleoyl-*sn-glycero*-3-phosphoglycerol (POPG) or 1,2-dipalmitoyl-*sn-glycero*-3-phosphoglycerol (DPPG), which is nearly 10% of the total lipids.^29^ Along with phospholipids, the LSM also includes roughly 8% weight-based cholesterol (CHOL)^29^ and about 8% weight–weight surfactant protein^32^ as well. The surfactant proteins are separated into two groups: hydrophilic surfactant proteins (SP-A and SP-D), which are present in the lipid bilayer, and hydrophobic surfactant proteins (SP-B and SP-C) that are embedded in the lipid monolayer as involved in the surface-tension controlling agent of the LSM.^33,34^

Asthma, rhinitis, and a few skin diseases can also be treated with the corticosteroid medication methotrexate (also known as mometasone furoate). Having anti-inflammatory, anti-pulmonary itching, and vasoconstrictive effects, mometasone is a synthetic glucocorticoid.^35–37^ Recent *in vitro* investigations have demonstrated that mometasone shows antiviral activity, which inhibits the replication of COVID-19 and MERS-CoV.^38,39^ Understanding the chemical interactions of the drug with LSM, which serve as the primary obstacle for inhaled particles to penetrate the alveoli, is crucial to comprehending the mechanism. By enhancing the drug's adsorption capacity into the pulmonary epithelium and alveolar interface, lung surfactants can be employed to increase drug efficacy and lower systemic toxicity. Hydrophobic drugs can also be dissolved with surfactants. The development of drug delivery mechanisms as well as the adsorption of the drug itself, depend on how drugs interact with the LSM. For instance, Cimato *et al.*^40^ investigated the interaction of beclomethasone, budesonide, and fluticasone with pulmonary surfactant. According to the results from electron resonance spectroscopy, the order of the phospholipids dropped as drug concentrations rose, leading to phospholipids accumulation in the monolayer for all three corticosteroids except cholesterol. In contrast to the three corticosteroids (fluticasone, budesonide, and beclomethasone), cholesterol shows the opposite impact on surface activity. The findings demonstrated that all steroids have lower surface tension (0–2 mN m^−1^) than cholesterol (6–8 mN m^−1^).^40^ The effects of corticosteroids on compressibility varied from those of cholesterol. The study also showed that there is a threshold dosage for transporting drugs without changing the biophysical behavior of the LSM.^40^ Based on these investigations and others^41,42^ on various corticosteroids and lung surfactant preparations, the interactions between corticosteroid and monolayer are complicated and dependent on the surfactant's composition, surface tension, physico–chemical characteristics, and drug dose. Davies *et al.*^42^ measured the adhesive force between the micro-sized corticosteroid budesonide and the surfactant monolayer by combining the atomic force microscope and Langmuir–Blodgett experiments. According to the study, surface pressure controls the hydrophobic connections between budesonide and the monolayer. They also find that high interfacial pressure (low surface tension) exhibits stronger interactions than low surface pressure (high surface tension). The author also observed that the hydrophobic drug budesonide interacts with the lipidic hydrophobic chain and influences membrane packing at high pressure. These experimental findings demonstrate that corticosteroid activity depends on concentration and is affected by surfactant composition, but the study fails to explain the dynamical surface activity of the drug or surfactant components as well as their bio-physical alternations at the molecular level. Keeping these fundamental concerns in mind, the molecular dynamics (MD) simulation can provide data on the behaviour of surfactant components such as lipids, proteins, and other small drug molecules on a molecular scale, which allows us to stipulate molecular level knowledge on the pulmonary surfactant and drug interaction.^43–45^ By employing *in vitro* (grazing incidence X-ray off-specular scattering, GIXOS) and *in silico* (CG MD simulation) techniques, Souza *et al.*^46^ investigated the interaction of flavonoids (naringin and naringenin) and DPPC containing lung surfactant to elucidate adsorption behaviour. Naringenin possesses anti-inflammatory and antioxidant effects. However, oral administration is challenging due to its limited oral absorption.^47^ As a result, a new delivery method was introduced to transport drugs directly into the alveoli alleviating lung inflammation. However, due to the simplification of the model (it only includes DPPC phospholipid per layer to form the monolayer, neglecting the other surfactant constituents such as POPC, POPG, CHOL), this study is unable to fully capture the lung surfactant properties. For this, it is further necessary to conduct research by incorporating POPC, POPG, CHOL that might mimic more realistic lung surfactant components and different drugs. Lung surfactant may be used as a drug transportation and spreading agent to treat preterm neonates and adults with pneumonia who have lung injury.^48^ It shows promise as a carrier for antibiotics, potentially improving drug delivery to the lungs and enhancing treatment efficacy for respiratory infections.^49^ However, the interactions between surfactants and antibiotics must be thoroughly evaluated to ensure the compatibility and effectiveness of the drug. This approach could lead to more efficient and safer treatments for lung infections. Using a combination of the Langmuir experiment, atomistic MD simulation, and PM-IRRAS research, Ortiz-Collazos *et al.*^50^ investigated the molecular interaction of levofloxacin with LSM made up of binary phospholipids (DPPC:POPC). They discovered that amphoteric levofloxacin stabilizes the LSM for a particular levofloxacin concentration of 10% w/w by expanding its area without altering the monolayer elasticity. Recently, we conducted a coarse-grained molecular dynamics study to inspect the impact of mometasone on the LSM consisting of lipids (DPPC–POPC–POPG), cholesterol and surfactant protein (SP-B and SP-C).^51^ The findings from this study showed that mometasone changes the monolayer's structure depending on the drug dose and induces LSM destabilization affected by interfacial tension and surfactant protein. At high drug concentrations, the ability of the drug to diffuse within the LSM is constrained, leading to monolayer collapse.

In the current study, the deformations of the drug-free and drug-containing surfactant monolayers have been investigated using the Langmuir experiments and coarse-grained molecular dynamics. It also explored how the monolayer alters mechanically under different mometasone furoate concentrations during inhale and exhale breathing. The phase analysis is explored by using the wider ranges of surface tensions outside the normal breathing surface tension. Using fixed APL simulations, monolayer phase behaviour and monolayer-to-multilayer spontaneous alternation at the liquid condense (LC) and liquid expanded (LE) phases have been examined. The underpinning monolayer model utilized in this study is made up of DPPC–POPC–POPG–CHOL (60 : 20 : 10 : 10) to replicate the lipid fraction of mature human lung surfactant in the alveoli.^52^ This quantity of surfactant fragments within the monolayer (in mol%) corresponds to the number of surfactant molecules (62% DPPC, 21% POPC, 11% POPG, 6% cholesterol (CHOL)) by mass.^52,53^

## 2 Materials and methods

### 2.1 Experimental procedure for Langmuir monolayer studies

#### 2.1.1 Materials and reagents

##### Materials

The phospholipid DPPC (≥99%), POPC (≥99%), and POPG-Na (≥98%), as well as cholesterol (≥99%) and mometasone furoate (*European Pharmacopoeia* standard) were collected from Sigma Aldrich (St. Louis, MO, USA). The monolayer-forming substances were dissolved in Chloroform Uvasol of spectroscopic grade purchased from Merck (Darmstadt, Germany). Stock solutions of concentration 1 mg ml^−1^ were mixed to obtain DPPC : POPC : POPG : CHOL, 60 : 20 : 10 : 10 mol% ratio. In experiments with the drug, an appropriate amount of mometasone furoate dissolved in chloroform was added. The hydrofluoric acid (HF, 48%) for silica wafers functionalization was collected from EMSURE (Merck, Darmstadt, Germany). The ultrapure water (18 MΩ cm, pH 6.25, TOC 1–3 ppb) was provided by the PureLab Classic UV system (ELGA, HighWycombe, UK) and used as a subphase in every measurement.

#### 2.1.2 Procedures of Langmuir trough experiments and atomic force microscope experiments

All Langmuir monolayer experiments were performed at the Teflon trough with two symmetrical barriers by KSV NIMA (Helsinki, Finland). The surface pressure (π, mN m^−1^) was measured with a platinum Wilhelmy plate with an accuracy of 0.1 mN m^−1^. The subphase temperature was kept constant by a Julabo F12 thermostat at 36.6 °C. The spreading solution was gently placed on the cleaned surface of the aqueous subphase and left for solvent evaporation for 10 min. To indicate the proper experimental conditions, we carried out preliminary isotherm experiments at several speed levels of sliding barriers. Based on those studies (Fig. S1†), the monolayer compression rate was set at all experiments for 10 mm min^−1^. As monolayer compression progressed, the surface pressure was recorded as a function of the average area per lipid [APL, Å^2^]. Based on the π-APL isotherm, the compression modulus (Cs^−1^, mN m^−1^) was determined according to the equation (eqn (1)):1
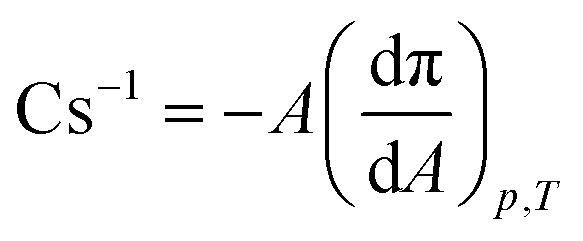


The compressibility of the monolayers was assigned to the state classification of the Davies and Rideal method.^54,55^ The relaxation experiments were employed to assess monolayer stability. The procedure was applied to compress the monolayer to 30 mN m^−1^, keep it constant and record the relative APL (*A*/*A*_0_) changing over time (*t*), where the *A*_0_ is the APL value when the initial pressure (π) is equal to 30 mN m^−1^. The surface potential (Δ*V*, V) was determined with the sensitivity of ±1 mV using the non-contact surface potential sensor SPOT, KSV Nima (Helsinki, Finland). The Δ*V*-APL isotherms were recorded simultaneously with the π-APL isotherms. The measurement involves the detection of the potential differences between the oscillating plate positioned just beyond the layer and the counter electrode submerged beneath the layer. The apparent dipole moment (*μ*_a_, D) was calculated as stated in the published experiment by using the equation (eqn (2)):^56,57^

2 *μ*_a_ = Δ*V*·*A*·*ε*_0_

where *ε*_0_ is the vacuum permittivity. Values of *A* and Δ*V* are extracted from experimental curves. Monolayers of DPPC : POPC : POPG : CHOL, 60 : 20 : 10 : 10 ratio compressed to various surface pressure levels were deposited onto freshly cleaved mica substrates (discs of 12 mm diameter). Systems mixed with various amounts of MF (0.72 and 4.18%) were transferred onto silica wafers functionalized in hydrofluoric acid (HF) to obtain a hydrophobic surface. Silica wafers were soaked in HF solution (2 ml of HF with 48 ml of ultrapure water) for 2 min, rinsed with ultrapure water, and let dry. The transfer was *via* the Langmuir–Blodgett (LB) approach. After 1 minute, when the surface pressure was reached and kept stable, the automated software-controlled dipper deposited film at a speed of 1 mm min^−1^ in an upward stroke. Films deposited on solid substrates were subjected to AFM (NX10, Park Systems, South Korea) tests up to 24 hours after the LB transfer. Collected data were post-processed in Gwyddion software. Using AFM, topography images, cross-sections, roughness, and average thicknesses of the different molecular components were acquired. The measurements in non-contact mode, at a nominal force constant of 40 mN m^−1^, were performed using the All-In-One D AFM cantilevers (Budget Sensors, Bulgaria). The scanning rate varied between 0.3 and 0.5 Hz, while the dimensions of the scan area size ranged from 0.5 μm × 0.5 μm to 15 μm × 15 μm, and in some cases even 60 μm × 60 μm. The mean roughness values (*R*_a_) were extracted for each sample from section 2 μm long on a 5 μm × 5 μm micrograph. The uncertainty was obtained from the standard deviation of at least ten individual measurements.

### 2.2 Molecular dynamics simulation approach

In this study, we used a coarse-grained method to simulate the lung surfactant system. For this, we first parameterized the corticosteroid drug (Mometasone furoate) by following the process of our previously published paper for mometasone^51^ by adding two extra beads into the side chain moiety of the steroid ring at the C-17 position (Fig. S3†). The bead structure of the drug molecule has been briefly illustrated in the ESI Section (S1.2).† After constructing the CG molecular structure of mometasone furoate, the partition coefficient of the drug molecules was evaluated by using the umbrella sampling (US) technique. The obtained partition coefficient is 10.29 showing the stable coarse-grained structure of the mometasone furoate drug molecule. We have found overestimated the partition coefficient of mometasone furoate (10.29) compared to the log *P* values from *A* log *P* (4.27) and ChemAxon (5.06), which are QSAR tools used to predict log *P* values. Thus, the free energy of partitioning is too negative for mometasone furoate. However, the relative partition coefficients for mometasone (7.97,^51^), prednisolone (5.10,^58^) and cholesterol (8.72,^59^), which we used as a reference, are correct. If we would correct the parameters of mometasone furoate to match the ones from QSAR tools, it would not be correct relative to mometasone. Therefore, the final optimized configuration (stable coarse-grained structure of the MF) and parameters of the MF were used for the molecular dynamics simulation of the LSM in a concentration-dependent manner. A range of drug concentrations (MF) was then considered for evaluating the effect of drug dosages in experiments and simulations. The monolayer system is designed with the number of drug molecules necessary to achieve the appropriate drug concentrations. The number of lipid molecules in the model and the corresponding molecular weights of all monolayer constituents are used in calculating the drug concentration according to the equation (eqn (3)).3
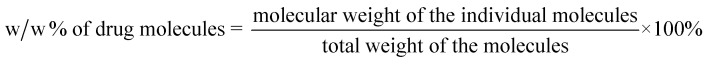


The number of drugs employed in the simulations is approximately the dosage that a meter dose inhaler delivers to the lungs per puff. The calculation is based on the usual adult lung volume, typical doses of anti-inflammatory drugs taken by inhalation, such as prednisolone, and the estimated 50% efficacy of pulmonary corticosteroid administration puffers.^60,61^ Corticosteroids are often administered to adults in doses ranging from 100 μg (for low dose) to 2000 μg (for high dose). The quantity of drug molecules needed for these doses can be determined using Avogadro's number and the molecular mass of mometasone furoate (521.40 g mol^−1^). The amount of the lung surfactant constituents and the make-up of the lung surfactant monolayer in the simulation system must therefore be taken into consideration. In the next paragraph, the lung surfactant monolayer system and the underlying simulation conditions have been discussed.

#### 2.2.1 Simulation systems setup

In this study, a reliable composition of surfactant monolayer components has been considered that corresponds to the amount of surfactant molecules, 62% DPPC, 21% POPC, 11% POPG, and 6% CHOL by mol%. The monolayer was built (Fig. S4†), maintaining the stated molar ratio of the surfactant molecules according to the protocol established by Hossain *et al.*^62^ and Islam *et al.*^51^ with the help of the Python script *insane.py*.^63^ The number of surfactant molecules was considered DPPC (1224), POPC (408), POPG (200), CHOL (200), and the number of water and ions were taken 47 546 and 6492, respectively. Five different models were constructed to simulate the drug containing LSM. Once getting the surfactant monolayer, there are several configurations of the monolayer that were created to reach constant area per lipid (APL) with the system size 220.0 × 220.0 Å^2^ (47), 223.1 × 223.1 Å^2^ (49), 227.7 × 227.7 Å^2^ (51), 232.2 × 232.2 Å^2^ (53), 236.5 × 236.5 Å^2^ (55), 240.8 × 240.8 Å^2^ (57), 245.0 × 245.0 Å^2^ (59) and 249.2 × 249.2 Å^2^ (61) as illustrated in Table 1. In the absence of a drug molecule, each of these eight systems (in System-I) was simulated 500 ns for constant particle number, volume, and temperature (*NVT*) equilibration where we monitor surface tension as a function time to verify the equilibrium state of the system and then 2 μs production run simulation (in the same *NVT* ensemble), twice. The MF molecules were arbitrarily placed in the air phase of the system (Fig. S5†). These five drug-containing systems were equilibrated for 500 ns followed by a 2 μs production run in the *NVT* ensemble for fixed APL (System I) simulation. A total of eighty simulations have been simulated to monitor the effect of drug concentration on monolayer compressibility.

**Table 1:** Drug (MF) concentration calculation for the monolayer models comprised of DPPC–POPC–POPG–CHOL (60 : 20 : 10 : 10) with a range of MF content (0 to ∼15.0% w/w). The models at fixed APL were employed at *NVT* ensemble in the absence and presence of a range (0 to ∼15.0% w/w) of drug molecules to compare the results with the Langmuir experiment

System	Drug concentrations (in % w/w)	System components	Drug concentrations (in mg)
Constant APL monolayer: APL = 47, 49, 51, 53, 55, 57, 59 and 61 Å^2^			
System-I	0%	DPPC–POPC–POPG–CHOL	0
	0.72%	DPPC–POPC–POPG–CHOL–MF	0.32
	2.13%	DPPC–POPC–POPG–CHOL–MF	0.95
	4.18%	DPPC–POPC–POPG–CHOL–MF	1.86
	8.02%	DPPC–POPC–POPG–CHOL–MF	3.58
	14.84%	DPPC–POPC–POPG–CHOL–MF	6.63

The molecular modelling software GROMACS version 5.1.4 (ref. 64) was used to simulate all the lung surfactant monolayer models. Each model was energy minimized using the steep descent algorithm. A 20 fs time step was considered for the leapfrog algorithm.^65^ The Martini standard cut-off distance was applied for the coulomb and Lennard-Jones interaction potential^66^ and polarizable water model.^67^ Monolayer components (lipids and cholesterol), water and ions, and MF were coupled independently at a temperature of 310 K with a *V*-rescale thermostat^68^ and a time constant of *τ* = 1.0 ps. The Berendsen barostat^69^ at temperature 310 K was used with *τ* = 4.0 ps. The neighbor lists were updated for each 20 steps. All the frames of the simulation from the last 1 μs of the final production run simulation were taken for data analysis, and the data were averaged over the duplicate runs. Data analyses were done with the help of GROMACS tools and Python scripts. For monolayer visualization, the VMD tool^70^ was used. The VMD compatible tcl script *cg_bonds-v5.tcl*^71^ was utilized for rendering the CG MARTINI bonds. To calculate the lipid order parameter, the Python script: *do-ordergmx5.py*^71^ was employed.

## 3 Results and discussion

### 3.1 Monolayer compressibility and stability analysis by *in vitro* experiment

**Fig. 1:**
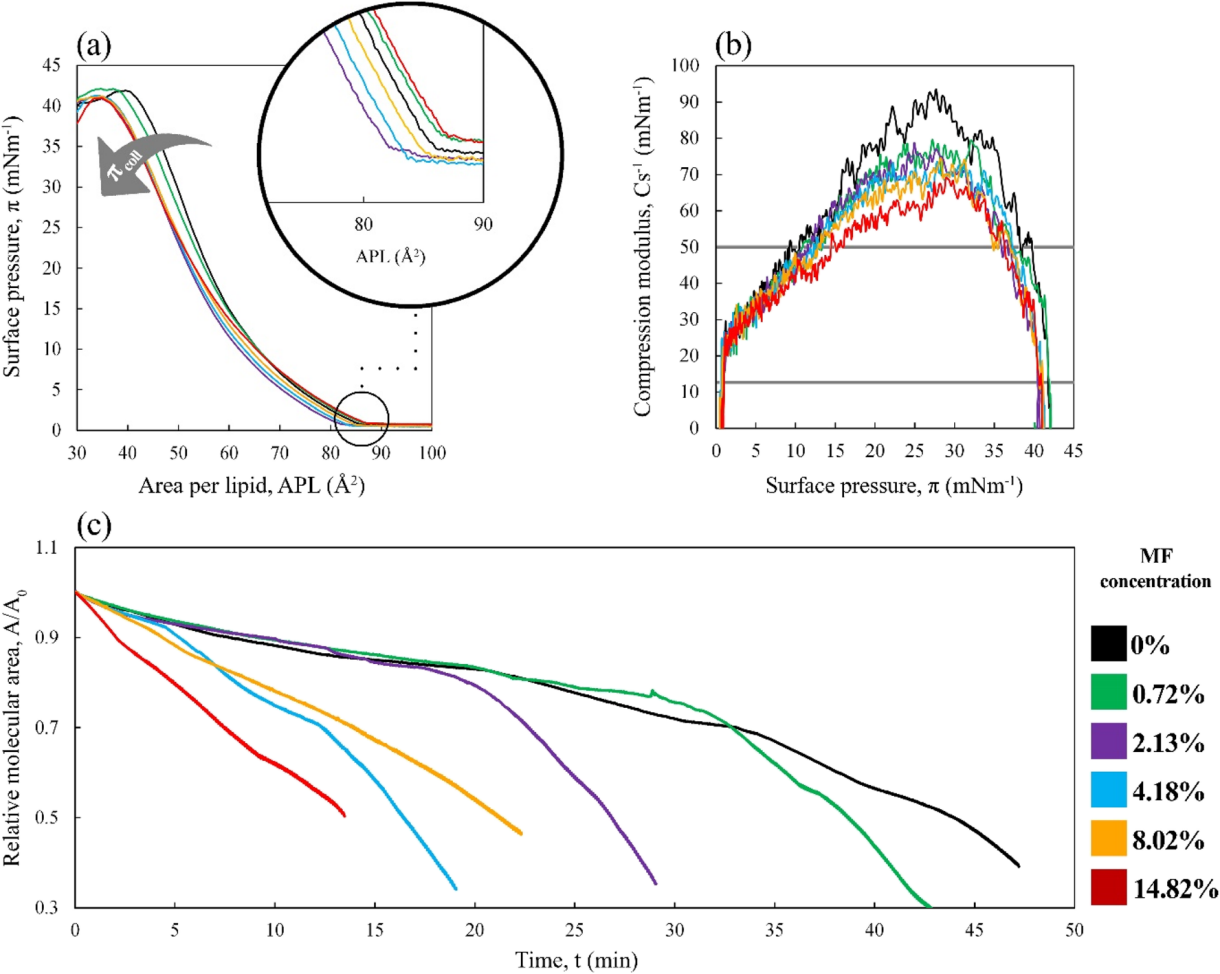
Effect of various concentrations of mometasone furoate on the mixed Langmuir monolayer of DPPC : POPC : POPG : CHOL, 60 : 20 : 10 : 10 at aqueous subphase at 36.6 °C, (a) the π-APL isotherms (inset): close-up of the initial experimental phase in the *A*_lift-off_ region, (b) the compression modulus *vs.* π, calculated based on the π-APL isotherms, (c) the relaxation experiments at a constant surface pressure of 30 mN m^−1^.

The isotherm curves as a function of drug concentrations have been calculated in the first part of the investigation to assess the compressibility and stability of the lung surfactant monolayer. When trying to understand how the monolayer behaves in terms of the monolayer phase during compression/expansion cycles, the π-APL isotherm is a crucial tool. Thermodynamical and structural details including the existence of different monolayer phase transformations such as liquid condensed (LC), liquid expanded (LE) or phase co-existence (LC + LE), can also be obtained from the compressibility parameters (compressibility modulus) of Langmuir monolayers determined from π-APL isotherms.^54^ The presence of mometasone furoate influences the shape of the π-APL isotherm for the lipid mixture DPPC : POPC : POPG : CHOL, 60 : 20 : 10 : 10 (Fig. 1a, S6 and S7†). The surface pressure (π) has been calculated from the surface tension of water (*γ*_a-w_ = 72.0 mN m^−1^ at 298 K (ref. 72 and 73)) and the surface tension (*γ*) of the mixed monolayer from the equation according to the study conducted by Sheridan Alan J., *et al.*^74^ and Hu Jiajie, *et al.*^75^

For the π-APL isotherm of the drug-free control system, the *A*_lift-off_, corresponding to an average area filled by the molecules over the monolayer when π increases above 0 mN m^−1^, is equal to 86 Å^2^ in the applied measurement conditions. In order to show the comparison between isotherm curves from the Langmuir experiment at different drug concentrations, similar π-APL isotherm curves were reproduced from MD simulation (Fig. S7†) according to the procedure employed by Baoukina *et al.*^76^ It is worth noting that simulations cannot cover the same extensive range of APL as used in Langmuir experiments due to the underestimation of interfacial tension from simulations (Fig. S6 and S7†). In applied thermal conditions (36.6 °C), the maximal surface pressure obtained by the monolayer of the phospholipid mixture with cholesterol is calculated 42 mN m^−1^. However, it is still feasible to assess the impact of the MF on APL in the range of APL from 47 to 61 Å^2^. The results from Langmuir experiments demonstrate that π is decreased by the increase of the MF concentration for the constant APL. This impact is apparent in our computations as well. The results of the experiment further demonstrated that for a fixed APL, the drug reduces the value of π in the monolayer, which may contribute to optimizing the breathing mechanics(Fig. 1a and S6†). This impact is still apparent in the *in silico* study, albeit less strongly than in the experiment (Fig. S7†). From Langmuir experiments, phase transitions cannot be distinguished in the course of isotherm in applied conditions, even though they are typical for the monolayer of DPPC (which dominates the mixture). For systems with various drug content, there is no evident relationship between the drug concentration and monolayer expansion (noted as the *A*_lift-off_ shifting toward larger values). As shown in the inset in Fig. 1a, the curve with the addition of 0.72% w/w MF is close to the isotherm of the drug-free, but the addition of 2.13% MF shifts the curve towards a lower value of APL. Considering that MF is not included in the average value of the area per lipid molecule, this indicates the reorganization of the monolayer molecules caused by the presence of the drug molecules so that they occupy a smaller space. The curves corresponding to the drug-free system, and systems containing 0.72% and 14.82% MF are indistinguishable for APL > 60 Å^2^. It has been found that MF concentration notably affects the isothermal slope. Therefore, the curve slope is more towards the *X*-axis with increasing drug content and collapses at the lower surface area. This phenomenon is reflected in the compressibility modulus (Cs^−1^) values (Fig. 1b). The maximal Cs^−1^ values calculated for all systems fit between 50 and 100 mN m^−1^ (an intermediate region between the LE and LC states). At the higher drug content in the mixture, the lower of the maximum Cs^−1^ value is observed. Mometasone furoate is insoluble in water, and the drug itself does not form a monolayer. This property may provide challenges in pharmaceutical formulations where solubility is essential to the effectiveness and delivery of drugs. However, the changed course of the π-APL isotherms and Cs^−1^*vs.* π curves indicate that MF molecules are present in the vicinity of the interface, not exactly occupying space on the surface but certainly altering the organization of the lipid monolayers. Since the presence of drug molecules lowers the surface pressure when the monolayer collapses in this case, we assessed the effect of MF on monolayer stability *via* relaxations at a constant surface pressure of 30 mN m^−1^ (Fig. 1c). In such an experiment, the monolayer is subjected to the stress from barriers sliding to keep the π at the desired level. In this case, it causes loss of the molecules from the surface, illustrated as a relative molecular area decline for each system including the drug-free system as shown in Fig. 1c. As the amount of the drug increases, the mixed monolayer becomes less stable, and the monolayer disruption rate enhances. The above-presented Langmuir technique results indicate that the addition of the drug in a dose higher than 2.13% w/w causes significant alterations in the characteristics of the lipid monolayer which is also verified by the *in silico* studies.

**Fig. 2:**
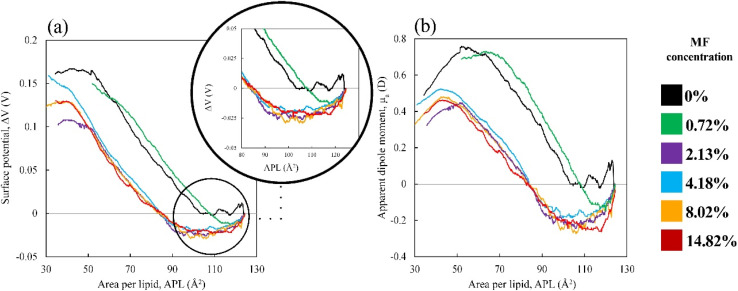
Effect of various concentrations of mometasone furoate on the mixed Langmuir monolayer of DPPC : POPC : POPG : CHOL, 60 : 20 : 10 : 10 at aqueous subphase at 36.6 °C, (a) the Δ*V*-APL isotherms (inset): close-up of the initial experimental phase, (b) the apparent dipole moment (*μ*_a_) *vs.* APL, calculated based on the Δ*V*-APL.

The influence of MF molecules on the mixed monolayers is noticeable since the initial stages of the monolayer formation, so the investigation should be broadened within this region. A surface potential-area per lipid (Δ*V*-APL) isotherms provide valuable insight into the molecular arrangement and packing when compressing a monolayer. The surface potential of a Langmuir monolayer relies on three primary factors: (i) the dipole moments of the monolayer components, (ii) the way water molecules are positioned towards each other, and (iii) the ionic environment as well as the condition of the head-groups and subphase.^77^ However, the crucial factor determining surface potential is the arrangement of charges across the surface. The surface potential arises from constant electric dipoles of the molecules developing monolayers and the reassembly of water molecules. A potential difference is created between the air and the negatively charged aqueous subphase due to the selective alignment of oxygen in the air phase and hydrogen in the aqueous subphase, leading to polarization of the air–water interface.^55^ The surface potential over large areas per molecule is typically zero. As the monolayer is compressed, and due to the domain's formation, reaches a critical area, the Δ*V* tends to increase with the reorientation of dipoles. Since this critical area is usually higher than the *A*_lift-off_ for the increase in surface pressure, the Δ*V*-APL isotherms are considered more suitable for monitoring the monolayer formation in the gaseous phase (when π is below 0 mN m^−1^) than the π-APL isotherms.^55,78^ The course of Δ*V*-APL isotherm of lipid mixture without MF follows the expected trend (Fig. 2a). Interestingly, the presence of the MF molecules entails a notable decrease in the Δ*V* value in the expanded molecular state (APL >110 Å^2^ for 0 and 0.72% MF and >85 Å^2^ for 2.13–14.82% MF). After that, the Δ*V* value increases sharply at a critical area almost identical for MF content above 0.72% w/w. Bearing in mind that the system studied here is very complex and no ions have been added to the aqueous subphase for the experiments, there are several possible explanations for such an outcome. Within the multicomponent lipid monolayer in the presence of the bioactive molecules, the decrease in Δ*V* value during monolayer compression can be attributed to increased intermolecular interactions, changes in molecular orientation, or electrostatic screening effects.

As a result of monolayer compression, the molecules are brought closer together, increasing intermolecular interactions, which may result in a redistribution of charges and a reduction in the overall surface potential. Compression can also cause a reorientation of the molecules within the monolayer. The altered orientation influences the dipole moments and distribution of charges and their exposure to the aqueous phase, thereby affecting the surface potential. Moreover, the decrease in intermolecular distance may lead to a partial neutralization of the charged head groups due to electrostatic screening by adjacent molecules, decreasing the overall surface potential. However, the lack of the Δ*V* drop in the initial compression stage for the mixed lipid monolayer and its intensification with increasing MF content proves that the presence of drug molecules in the interfacial region is crucial. As the permittivity of the monolayer (*ε*) is an unknown value, the changes in the effective dipole moment of the molecules within the monolayer due to compression were also presented as the apparent dipole moment (*μ*_a_) (Fig. 2b). As follows from the *μ*_a_-*A*PL isotherm, MF not only alters the surface potential in the expanded molecular state during the initial phase of the monolayer compression when MF molecules may move towards the hydrophilic regions of the monolayer or under the monolayer, but it affects the overall morphology of the film. It has been demonstrated by the significantly lower value of the maximal apparent dipole moment achieved by the MF-containing monolayers and the low values of the APL at the maximum *μ*_a_ value. Transforming the data into *μ*_a_ highlighted the maximum in apparent dipole moment values.

From the outcome of the Langmuir technique investigations, it is possible to conclude that MF molecules are present in the vicinity of the monolayer modifying the monolayer characteristics. However, the conclusions that could be drawn are insufficient to determine their exact position relative to the monolayer. Thus, monolayers were transferred onto a solid substrate and subjected to AFM studies to obtain topographical data. Fig. S8† summarizes AFM topographical images and cross-sections of mixed monolayers at different surface pressure levels to visualize the MF concentration-dependent effect. The cross-sections outline the topography of the characteristic elements. An AFM micrograph of the basic lipid system reveals the nano-domains with an average diameter of 39.88 ± 3.28 nm (measured at a picture in higher magnification) and height of 1–1.6 nm evenly distributed over the entire sample area. At an increased surface pressure of 20 mN m^−1^, the diameters are about 79.95 ± 16.67 nm, while at 30 mN m^−1^, it is 73.60 ± 11.69 nm with a height of 0.9 nm for both. The domains are still evenly distributed but more varied in size. A striking alteration in film topography is noticed as the π value increased to π_coll_. Due to the tight packing of the layer, the irregularly shaped domains merge into clusters. An analogous situation has been described for the system of DPPC : POPC : POPG, 50 : 30 : 20 at 50 mN m^−1^, but a continuous network was created there.^79^ It is worth noting that the larger clusters seen near the edge of the sample are probably not a region in a different phase because it is the same height (0.8–1.4 nm) as the smaller ones.^80^ No multilayers are observed for the lipid mixture. With the increasing surface pressure, the roughness values (*R*_a_) for the lipid film change insignificantly but are higher for the sample at the π_coll_ (Table 2).

**Table 2:** Roughness (*R*_a_) values obtained from AFM micrographs. For samples containing the MF (columns b and c), *R*_a_ values refer to the framework film area excluding visible aggregates

		Roughness [pm]		
		(a) 0% MF	(b) 0.72% MF	(c) 4.18% MF
I	10 mN m^−1^	54.5 ± 9.3	273.8 ± 34.8	320.9 ± 23.5
II	20 mN m^−1^	55.5 ± 14.5	216.1 ± 35.0	354.6 ± 45.0
II	30 mN m^−1^	57.2 ± 13.7	458.1 ± 68.8	282.4 ± 16.6
	**π**_**coll**_	117.0 ± 15.4	—	—

The presence of a drug concentration even as low as 0.72% w/w introduces noticeable alterations within the monolayer as the irregularly shaped, usually multi-leveled aggregates of the maximal height of 12–20 nm at 10 mN m^−1^. In the presence of 4.18% MF, there are fewer aggregates, but they are of a larger area and a height of up to 30 nm. However, it has been noted that the topography of the film between the aggregates resembles that of a drug-free lipid monolayer. The relative height differences of the structures from the cross-sections in the areas without aggregates are approx. 0.8, 0.9, and 2.3 nm for samples at 10 mN m^−1^ as presented in the first row of Fig. S8a, b and c,† respectively. The cross-section comparison is given in Fig. S9,† and the roughness values (*R*_a_) for the film framework area, excluding visible aggregates, are in Table 2. With the increasing MF content, the *R*_a_ values for the flat framework are higher than for the MF-free film. However, values calculated from the top of the aggregates exceed the values of aggregates-free regions by order of magnitude, reaching 1640 ± 752 pm and 1920 ± 691 pm for the I-b and I-c samples, respectively (data not shown). Aggregates in systems containing 0.72% w/w of the drug in elevated π are of the same height, but at 20 mN m^−1^, it is more numerous, and at 30 mN m^−1^, they group into structures of a large area. A significant proportion of the drug in the system (column c) contributes to the further increase in the relative height of the aggregates. Until, at 30 mN m^−1^, next to the large-area aggregates there are also lower structures resembling those of the lipid monolayer in π_coll_ but of a much greater height. From the experiments that have been undertaken at the air–water interface, it is possible to conclude that MF molecules associate with the lipid head group compared to the lipid tail in response to the monolayer compression above the surface pressure of 10 mN m^−1^ (transition from the LE phase to the intermediate phase between LE and LC). That is noticeable in the course of isotherms and the Cs^−1^ modulus. The presence of MF molecules under the monolayer affects its characteristics and decreases stability by altering the orientation of lipid molecules. As the monolayer is further compressed, the MF molecules form aggregates of considerable thickness. Interestingly, the BAM (Brewster angle microscope) images were also performed for the lipid system and one containing significant amounts of MF (data is shown in Table S3†). In both cases, only foam-like structures characteristic of lipid mixtures were observed for the expanded monolayer. The lack of aggregates may indicate the presence of drug molecules only under the lipid monolayer. Moreover, the basic lipid system was transferred by the LB technique to the mica substrate, which is hydrophilic. However, the transfer of the drug-containing layer to the same substrate has failed. Thus, in the subsequent steps, a successful transfer was made onto a hydrophobized silicon substrate. During the transfer of the monolayers onto the flat solid surface, the aggregates could be emphasized, which is visible in the AFM studies. It has been demonstrated that, as the drug content of the monolayer increases, its compressibility and stability over time decrease. Unfortunately, based on the performed tests, it cannot be stated whether the MF molecules are present on the surface in an expanded monolayer.

### 3.2 Monolayer structural arrangement and dynamical behavior analysis by *in silico* investigation

**Fig. 3:**
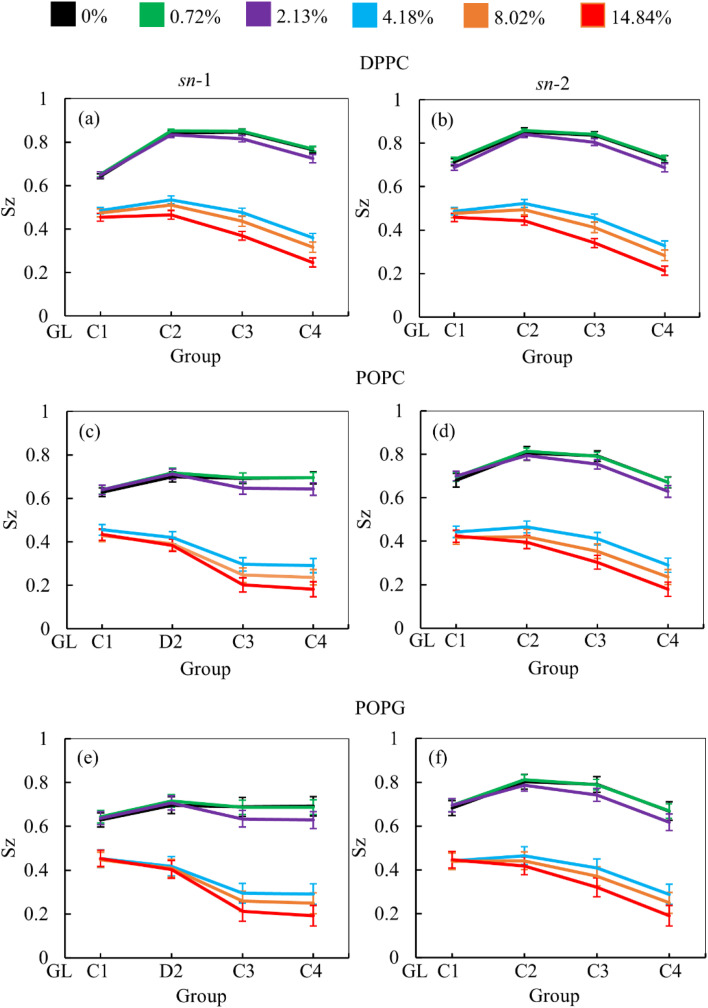
Order parameters of the lipids; DPPC (a and b), POPC (c and d), and POPG (e and f) for *sn*-1 and *sn*-2 chains in columns at increasing drug concentrations for the APL value 47 Å^2^. The values were calculated for the last 1 μs of the 2 μs simulations. The standard deviations were estimated using the frames of trajectories.

We used computational modeling to determine the properties of the monolayer and the drug diffusion. To assess the effect of drug molecules on the monolayer lipid components (DPPC, POPC, POPG) at highly condensed (47 Å^2^) and expanded (61 Å^2^) surface of the monolayer, the lipid order parameter was estimated as a function of MF concentration with the help of *in silico* simulations. The order parameter value ranges from −0.5 to 1.0, with −0.5 indicating complete anti-alignment along the monolayer normal and 1.0 indicating perfect alignment with the monolayer normal. Fig. 3 reveals that the lipid tail (chain-1, *sn*-1 and chain-2, *sn*-2) orientation is aligned to the normal of the monolayer surface at highly condensed (47 Å^2^) monolayer at increasing drug concentration from 0 to 14.84% w/w. The overall comparison of the lipid chains (*sn*-1 and *sn*-2) demonstrates that all the saturated tails (DPPC *sn*-1, DPPC *sn*-2, POPC *sn*-2, and POPG *sn*-2) follow a similar trend whereas, the unsaturated tails (POPC *sn*-1 and POPG *sn*-1) show a different orientation compared to saturated tails (Fig. 3). These dis-similar orientations between saturated and unsaturated lipid chains are because of the C–C double bond at D2–C3 bead of the lipid chain. It is typically known that at lower molecular area, the lipid chain should be highly ordered, but for unsaturated chains, the order parameter declines compared to saturated chains. This effect has also been observed for all the investigated drug concentrations.

**Fig. 4:**
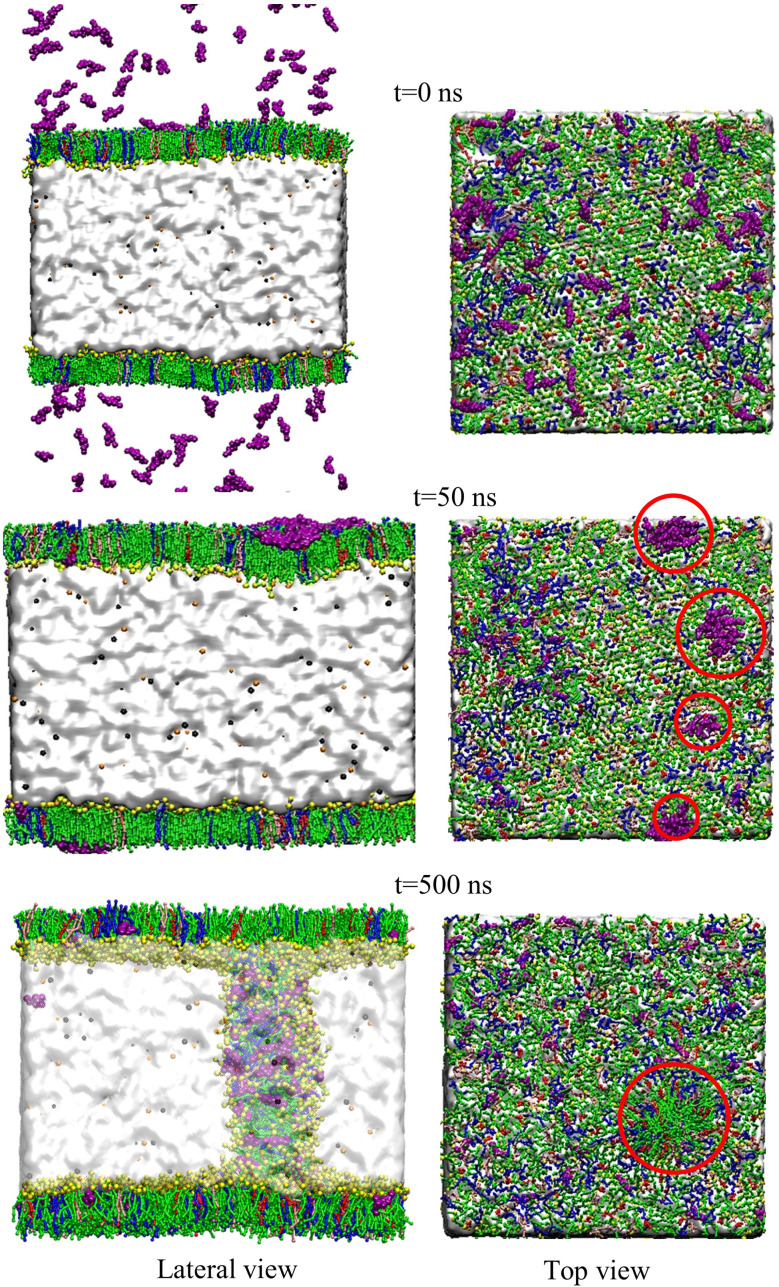
The collapsing mechanism at APL value of 47 Å^2^ for the drug concentration 4.18% w/w. The snapshots were captured at different simulation times from both the lateral and top view of the monolayer. DPPC is pointed by green, POPC in blue, POPG in pink, cholesterol in red, MF in purple, water in white, Na^+^ in orange, and Cl^−^ in black as well as lipids head group in yellow.

At concentrations up to 2.13% w/w, the lipid chains are highly aligned with the monolayer normal, but a substantial drop of order parameter has been observed when the drug concentration exceeds 2.13% w/w. The massive amount of MF molecules disrupts the lipid monolayer which induces collapse (Fig. 4). Along with the change in the liquid-ordered phase of the monolayer, the drug molecule moves into the low molecular area and starts accumulation inducing the system to break down.^81^ As opposed to the order parameter at the highly condensed monolayer (47 Å^2^), the order parameter at the extended state (61 Å^2^) illustrates the alternative situation, in which the order parameter rises as drug concentrations rise. Due to the maximum APL tested, the tails of the lipids are exhibiting low order (<0.6) as shown in Fig. S10.† These increasing orders of the lipid tails are noticeable for beads C1–C2/D2 and C2/D2–C3 for all the lipids, but for the C3–C4 bead, there is no significant alteration that occurred. Due to the passive drug diffusion, the accumulation of drug molecules near the lipid head group causes higher order compared to the tails group, which is confirmed by the RDF calculation (Fig. 6 and S12†). Such patterns of the lipid order parameter also provide phase behavior information such that the monolayer is going to an intermediate phase termed phase co-existence (*e.g.*, LC–LE phase) from lower APL to higher APL, which is highly influenced by the drug molecules.

**Fig. 5:**
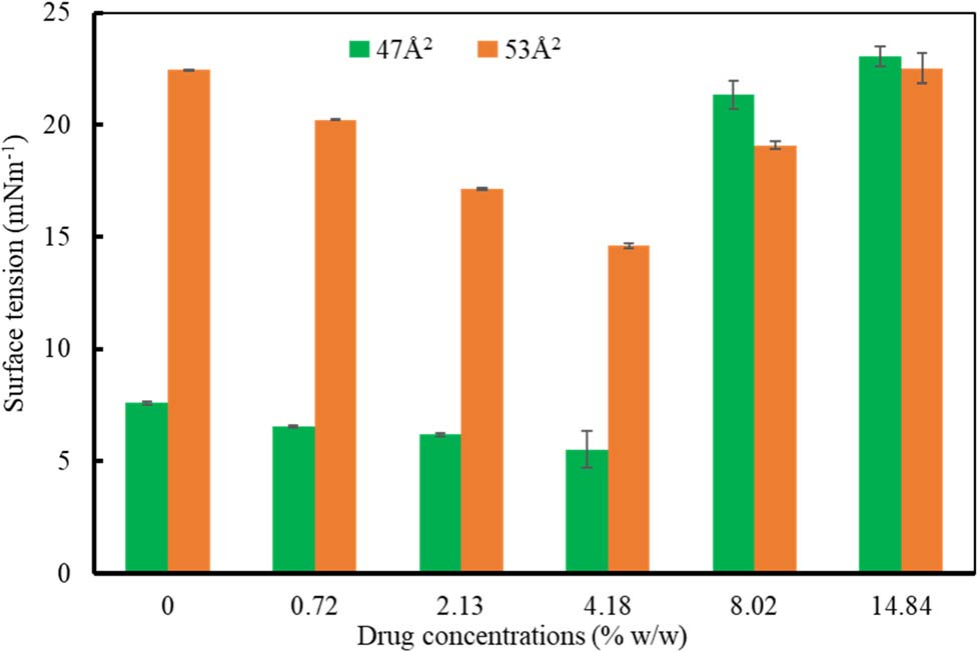
Impact of drug concentration on the surface tension at constant APL values of 47 Å^2^ and 53 Å^2^. A low APL value (47 Å^2^) refers to the molecular area at the exhalation breathing condition and a high APL value (53 Å^2^) indicates the molecular area at the inhalation breathing condition. The estimated errors using block averaging are shown as error bars.

In order to isolate the impact of drug concentrations on the surface tension of lung surfactant monolayer, the simulations were repeated at two different values of APL (47 Å^2^ and 53 Å^2^) for the drug concentrations from 0 to 14.84% w/w as illustrated in Fig. 5. The low APL value (47 Å^2^) refers to the molecular area at the exhalation breathing condition and the high APL value (53 Å^2^) indicates the molecular area at the inhalation breathing condition. For a drug concentration up to 4.18% w/w in both APL values, the surface tension of the monolayer follows a similar decreasing trend, but above the concentration of 4.18% w/w, the monolayer exhibits the opposite scenario, where the surface tension increases as the drug concentration exacerbates. At the low APL (47 Å^2^), the monolayer replicates the reduced surface tension (<7 mN m^−1^) that is observed at the end of an expiration breathing condition in the absence of a drug.^27^ The drug molecules then stimulate the monolayer to further reduce the surface tension (<5 mN m^−1^) up to the drug concentration of 4.18%.

As opposed to the APL value 47 Å^2^, the higher APL value (53 Å^2^), the surface tension is determined to be 23 mN m^−1^ (drug-free monolayer), which demonstrates that the monolayer can accurately reproduce inhalation breathing.^27,82^ The surface tension of the monolayer further falls from 23 mN m^−1^ to 15 mN m^−1^ when drug concentration rises from 0.72% to 4.18% w/w. It means that at both APL values, the drug molecules affected the surface tension of the lung surfactant to keep healthy breathing (maintaining the surface tension between 0 and 25 mN m^−1^), which is also supported by previous studies.^51,80^ However, when the drug concentration exceeds 4.18% w/w, the monolayer is disrupted by pore formation (at maximum drug concentration 14.84%, Fig. S13†) or monolayer collapse as a result of the imbalance in the surface tension at both APL values (Fig. 5). Such behavior is also seen in previous studies.^81^

Drugs may also aggregate in the form of aerosol droplets when it is in the air before entering into the monolayer. The lipophilicity or hydrophobicity of the drug molecule induces the construction of nano-accumulation of surfactant molecules on the monolayer. Due to the drug–drug adhesive force and drug–lipid cohesive force in the system, such aggregation of MF molecules is observed on the monolayer causing monolayer instability at higher drug concentrations at low molecular areas (Fig. 4).

**Fig. 6:**
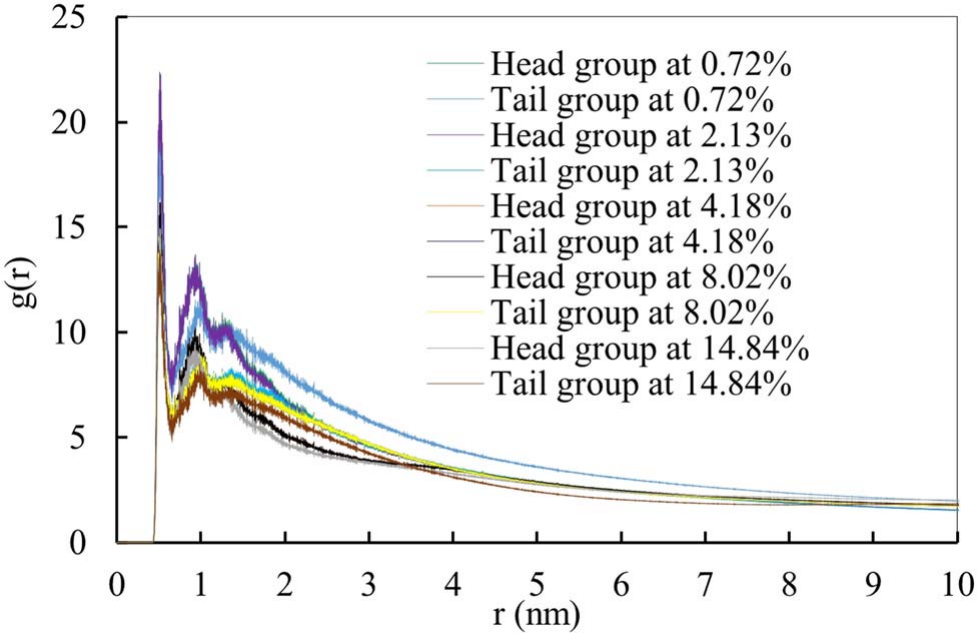
The radial distribution function (RDF) between drug molecules and lipid monolayer (lipid head and lipid tails) at different drug concentrations within a cutoff distance of 1.0 nm during lung compression (APL value of 47 Å^2^).

To analyze such accumulation or spreading activity of the MF molecules over the monolayer, the radial distribution function or pair correlation function, *g*(*r*) was computed by applying the cutoff distance of 1.0 nm. The variations in interaction mechanism between drug–lipid head and drug–lipid tail have been illustrated as a function of drug concentrations in Fig. 6 (for compressed monolayer) and Fig. S12† (for expanded monolayer). Mometasone furoate molecules prefer to interact with the lipid head compared to the lipid tail which is highly influenced by the higher concentration of the drug molecules. The drug molecules flee away from the hydrophobic layer formed by the lipid tail group and penetrate into the lipid head group region at the time of interaction. Therefore, the drug molecules are influenced to self-aggregate during the monolayer compression and induce the monolayer instability causing the monolayer collapse (Fig. 4). The opposite scenario can be noticed during the monolayer expansion (Fig. S11†) with proper spreading of the drug molecules over the monolayer surface. This is because of the balancing of the surface tension at inhalation (∼20 mN m^−1^) as well as exhalation breathing condition (<5 mN m^−1^), respectively (Fig. 5). Due to the larger molecular area available for the lung surfactant lipids at breath-in condition, the drug molecules can penetrate into the monolayer easily and contact with the choline group of the lipid head group by cationic–π interaction. As a result, the monolayer might be stable and the drug could be homogeneously distributed into the lipid structure, that maintains proper surface tension of the alveolar air–water interface. On the other hand, during the exhalation breathing condition, the monolayer components get lower space to move, and the drug molecules can interact with lipid head adjacent water molecules that might cause an increase of interfacial surface pressure. This high surface pressure may induce monolayer collapse at higher drug concentrations. The drug molecules and lipid head groups are then forced out from the monolayer air–water interface. This is because of the variations in the lipid head groups' current state on the aqueous surface by reducing the cation–π interaction.

## 4 Conclusion

In this work, the Langmuir experiment and MD simulations were employed to examine the dose-dependent impact of the corticosteroid drug mometasone furoate on cholesterol-enriched lung surfactant monolayer. It demonstrates that MF molecules influence the characteristics of DPPC–POPC–POPG–CHOL monolayers as well as the location and distribution of MF molecules in LSM with different surface pressures. Drug molecules can impede the development of liquid-condensed films and reduce the compressibility of mixed lipid monolayers. The cationic–π interactions between the choline molecule of the lipid head and the benzene ring of the MF molecule, the steric resistance of the lipid head, and the lipophilicity of the MF molecule all have an impact on the location and spreading of MF molecules into the lipid monolayer. There were more intermolecular interactions, changes in molecular orientation, or electrostatic screening effects inside the multicomponent lipid monolayer containing bioactive molecules as a result of the decrease in surface potential difference during monolayer compression. Lower surface pressures allow the cation–π interaction to take place, which causes drug molecules to combine with lipid choline molecules and make direct contact with water. Higher surface pressure at lower molecular area causes head groups of lipids to have close contact with water, resulting in the formation of a denser monolayer which is verified by the lipid order parameter from the computational study. To be transported into the aqueous phase, the drug molecules must overcome a stronger barrier. Drug molecules cannot be further transported to the aqueous phase by the stiffer monolayer. In line with this, the drug molecules that were initially connected with the lipid head groups are extracted from the layer. The drug molecules can escape from the lipid head group and tend to self-aggregate by forming bigger species when surface pressure intensifies. Drug molecule aggregation at high concentrations inhibits drug diffusion into the lipid monolayer, leading to the monolayer collapse. This drug-induced collapse of the monolayer is particularly likely to occur during exhalation breathing. Because of their hydrophobicity, these molecules then linger in a more hydrophobic microregion of lipid films. This study not only offers a potential mechanism of interaction between lipid complex monolayers and drug molecules but also brings in additional detailed information to help further understand the process of inhaled administration therapy. The overall findings of this investigation could advance our knowledge of how inhaled corticosteroid drugs interact and control the off-target drug delivery mechanism.

## Data availability

All data are presented in the paper in either figure or tabular form. The data is also provided in the ESI.†

## Author contributions

M. Z. I. developed the study concept and model, performed the simulations and formal data analysis, and drafted the original manuscript. M. K. conducted the experiment, drafted the experimental part of the original manuscript, and participated in data analysis. K. P. supervised the experimental part of the study and reviewed the manuscript. S. C. S. helped with data analysis and edited the manuscript. All authors gave final approval for publication and agreed to be held accountable for the work performed therein.

## Conflicts of interest

The authors declare that they have no known competing financial interests.

## Supplementary Material

RA-015-D5RA00004A-s001
