# Supplementary material for: In vitro and in silico studies of the interaction between glucocorticoid drug mometasone furoate and model lung surfactant monolayer
Source: RSC Adv. 2025 Feb 26;15(8):5951–64. doi: 10.1039/d5ra00004a (PMC11862883; doi:10.1039/d5ra00004a)
Supplement: RA-015-D5RA00004A-s001 [file RA-015-D5RA00004A-s001.pdf]

Supplementary Information (SI) for RSC Advances.
This journal is © The Royal Society of Chemistry 2025

Supplementary information:

In Vitro and *In Silico* Studies of the Interaction between Glucocorticoid Drug Mometasone Furoate and Model Lung Surfactant Monolayer

Md. Zohurul Islam[1,2, a], Martyna Krajewska[3], Krystyna Prochaska[3] and Suvash C. Saha[4]

[1]*Department of Mathematics, Faculty of Science, Jashore University of Science and Technology, Jashore-7408, Bangladesh*
[2]*High Performance Computing (HPC) Laboratory, Department of Mathematics, Jashore University of Science and Technology, Jashore-7408, Bangladesh*
[3]*Institute of Chemical Technology and Engineering, Poznan University of Technology, Berdychowo 4, 60-965 Poznań, Poland*
[4]*School of Mechanical and Mechatronic Engineering, University of Technology Sydney, 15 Broadway, Ultimo, NSW 2007, Australia*

[a] Corresponding author: Md. Zohurul Islam (mz.islam@just.edu.bd)

Table S1: Composition of lung surfactants used in different experimental and computational studies.

Experimental study	Computational study	Present study	Reference
DPPC	-	-	Ortiz-Collazos *et al.* [1]
DPPC: DPPG (4:1)	DPPC: DPPG (4:1)		Hu, Jiajie *et al.* [2]
-	DPPC: POPC (7:3)	-	Estrada-López *et al.* [3]
DPPC: POPC (7:3)<br>DPPC: POPC: CHOL (7:3:1)<br>DPPC: POPC: POPG (7:3:3)<br>DPPC: POPC: POPG:HOL (7:3:3:1)	DPPC: POPC (7:3)<br>DPPC: POPC: CHOL (7:3:1)<br>DPPC: POPC: POPG (7:3:3)<br>DPPC: POPC: POPG:HOL (7:3:3:1)	-	Islam *et al.* [4]
-	DPPC: POPC: POPG:HOL:SP (60:20:10:10:1:1)	-	Islam *et al.* [5]
-	DPPC:POPG (7:3)<br>DPPC:POPG:DOPC (3:1:1)<br>DPPC:DOPC:CHOL (5:3:4)<br>DPPC:POPG:DOPC:PA:SP (5:2:2:1:1)	-	Stachowicz-Kuśnierz *et al.* [6]
-	DPPC:POPG:CHOL:SP (70 :30:10:1)	-	Hossain, S. I. *et al.* [7]
DPPC:POPC (70:30)	DPPC:POPC (70:30)	-	Ortiz-Collazos *et al.* [8]
-	-	DPPC: POPC: POPG:CHOL (60:20:10:10)	-

S1.1 Experimental methodology

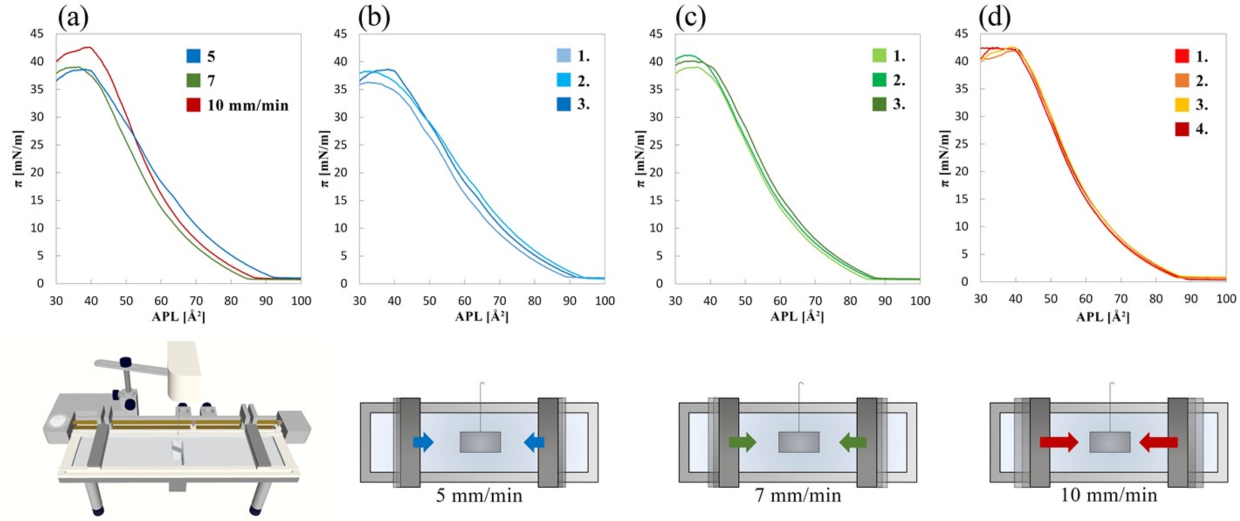

Figure S1: Determining the barrier speed for further measurements at aqueous subphase of 36.6°C, (a) comparison of the π-APL isotherms for DPPC:POPC:POPG:CHOL, 60:20:10:10 system for all barrier speeds tested and repeatability of the π-APL isotherms at (b) 5 mm/min, (c) 7 mm/min, and (d) 10 mm/min.

S1.2 Bead structure for mometasone furoate

The chemical structure of the mometasone and mometasone furoate has been illustrated in Fig. S2, and the carbon number of the sterol core has been shown to identify the position of the side chain moiety of the drug molecules. The modeling of the mometasone furoate CG model has been given and shown in Table S2. This is performed by the small molecule parameterization method from the CG Martini modeling [9, 10]. The bead mapping of mometasone furoate was structured as shown in Fig. S3 according to the bead skeleton of mometasone [3]. The partitioning free energy was determined via umbrella sampling simulation after obtaining a stable CG mometasone furoate model according to the protocol conducted by Islam *et al.* [5] with two extra beads into the side chain moiety of the steroid ring. The partition coefficient (logP value) of mometasone furoate was calculated from the Gibbs free energy difference to confirm the mometasone furoate CG parameter. After that, the calculated value of the drug was compared to the partition coefficient of mometasone furoate with predicted and other simulated results [3, 11, 12]. It is crucial to note that while mometasone furoate's experimental logP value for lung surfactant monolayer has not yet been reported in the literature, the parameterization of this model was validated using mometasone furoate's octanol-water partition coefficient (logP). The obtained logP value is also compared to anticipated logP values at various scales from the mometasone furoate drug data bank [13, 14].

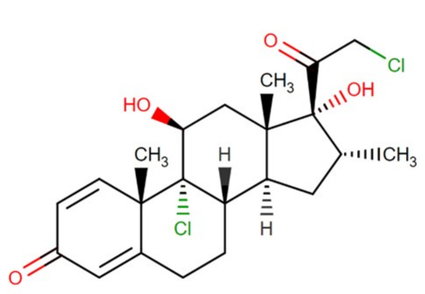

(a) Mometasone

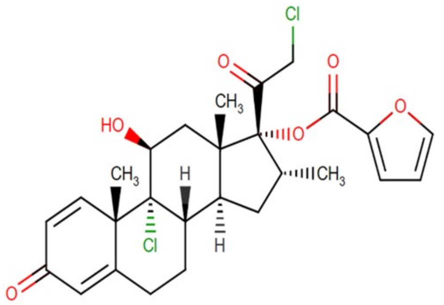

(b) Mometasone Furoate

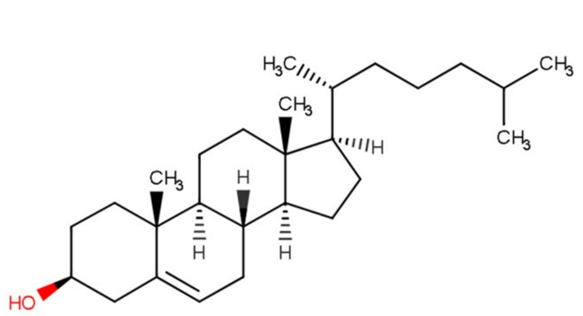

(c) Cholesterol

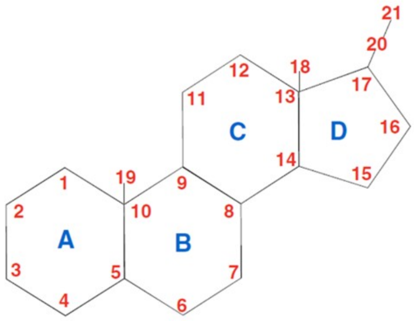

(d) Sterol core

Figure S2: Chemical structure of the (a) mometasone, (b) mometasone furoate, (c) cholesterol, and (d) the carbon number of the sterol core to show the position of the side chain moiety of the drug molecules and cholesterol.

Table S2. Detailed information about the CG model of the Mometasone furoate and the Mometasone drug.

Molecule	Bead name	Molecule name	Bead type	Group representation
	1B	RO1	SNa	Cyclic ketone
	2B	R1	SC4	Cyclic diene
	3B	RCL1	SC3	Cyclic chloro-alkane (RCL)
Mometasone furoate	4B	ROH	SP1	Cyclic alcohol
	5B	R2	SC2	Cyclic alkane
	6B	R3	SC2	Cyclic alkane
	7B	RO2	SNa	Cyclic ketone
	8B	RCL2	C3	Aliphatic chloride
	9B	ROO	Na	Aliphatic acetic acid
	10B	RO3	SNa	Cyclic Propanal
	1B	RO	SNa	Cyclic ketone
	2B	R1	SC4	Cyclic diene
	3B	RCL	SC3	Cyclic chloro-alkane
Mometasone	4B	R2OH	SP1	Cyclic alcohol
	5B	ROH	SNda	Cyclic alcohol
	6B	R3	SC2	Cyclic alkane
	7B	RO	SNa	Cyclic ketone
	8B	R4CL	C3	Aliphatic chloride

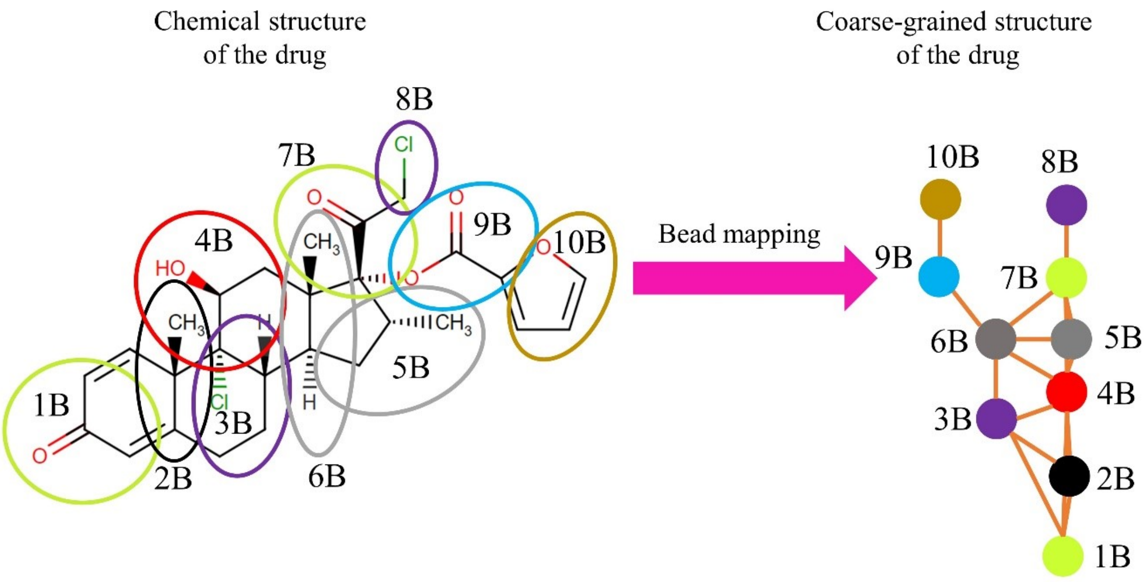

Figure S3: Coarse-grained bead mapping of the mometasone furoate. A group of atoms (indicated by a colored ring) has been considered a single heavy atom known as the coarse-grained bead.

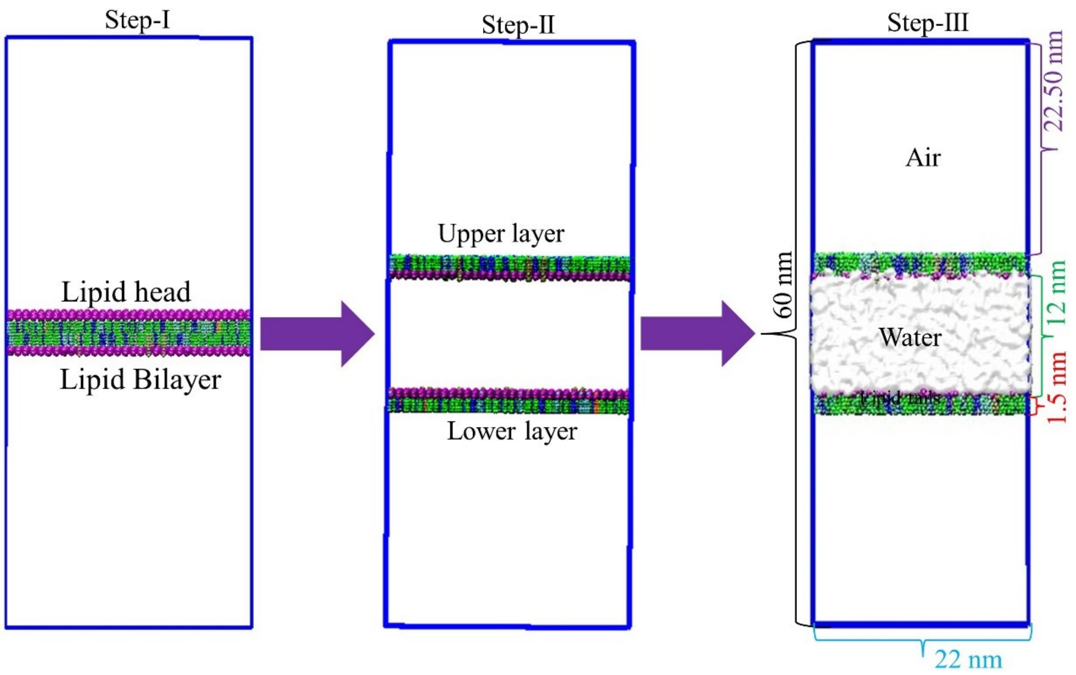

Figure S4: The step-by-step procedure of LSM formation from lipid bilayer to monolayer by maintaining the lipid mixture DPPC-POPC-POPG-CHOL (60:20:10:10).

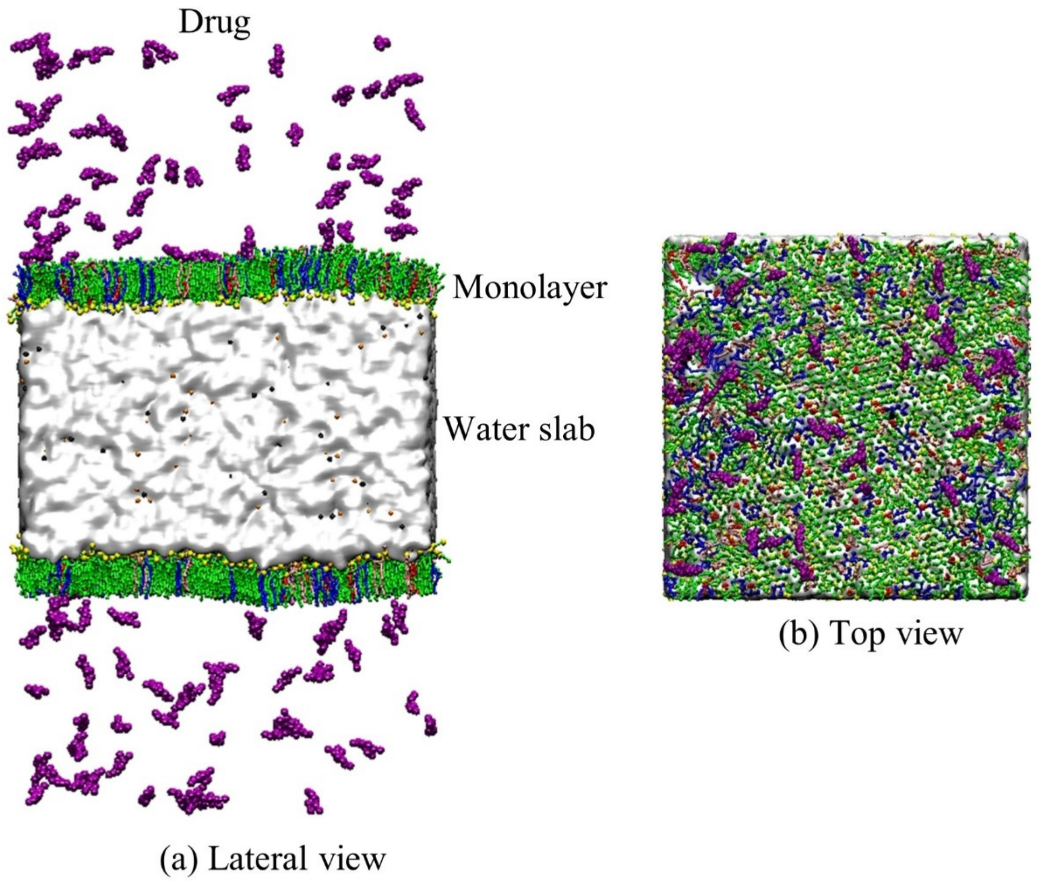

Figure S5: The drug-containing monolayer (a) Lateral view of the monolayer and (b) top view of the monolayer. The drug molecules were randomly placed over the monolayer surface at a certain concentration.

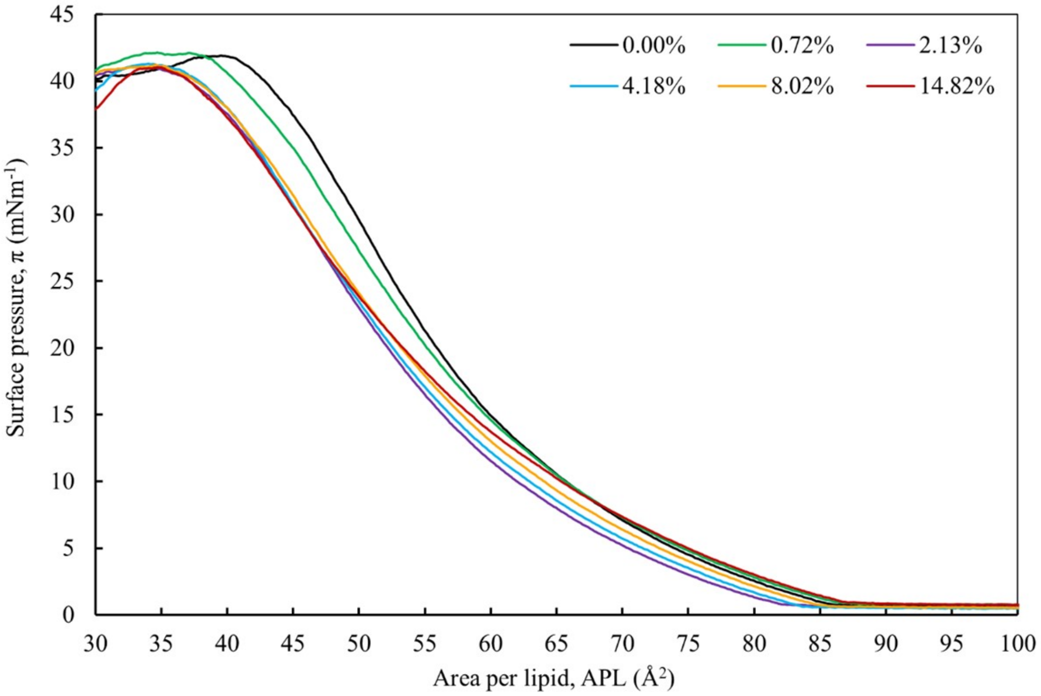

Figure S6: Surface pressure-area per lipid (π-APL) isotherms from Langmuir experiment for the mixture of DPPC-POPC-POPG-CHOL (60:20:10:10 mol %) with increasing drug concentrations (310 K, water subphase).

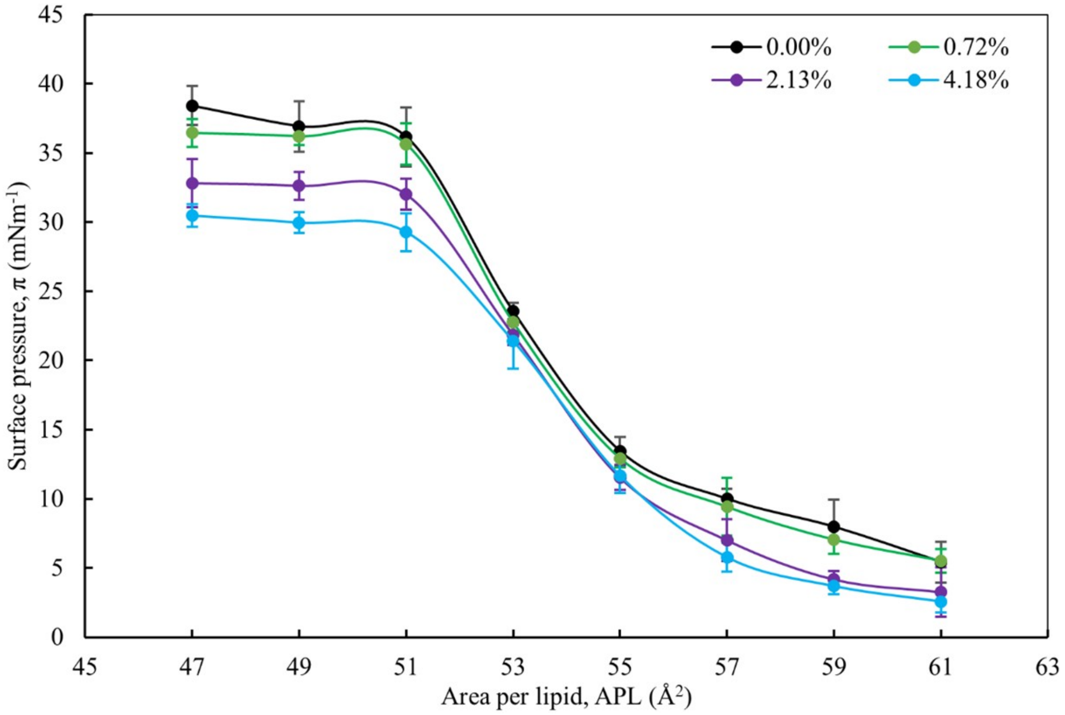

Figure S7: The pressure-area (π-APL) isotherms calculation from MD simulation of cholesterol-containing lung surfactant monolayers (DPPC-POPC-POPG-CHOL, 60:20:10:10 mol %) for increasing drug concentrations (mometasone furoate) to determine the impact of monolayer compression on the organization of the lateral structure of the lung surfactant monolayer.

Table S3. BAM (*Brewster Angle Microscopy*) images captured during compression of the π-APL isotherms for (a) a drug-free system of DPPC-POPC-POPG-CHOL (60:20:10:10 mol%) and (b) a system containing 14.82% Mometasone furoate. The surface pressure at which the images were captured is indicated below.

**(a) 0% MF**	
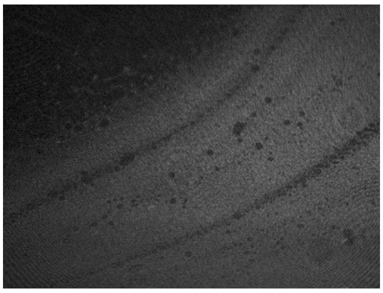<br>0.55 mNm^{-1}	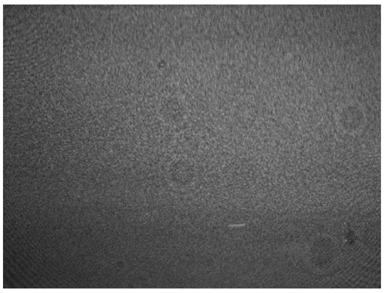<br>9 mNm^{-1}
**(b) 14.82% MF**	
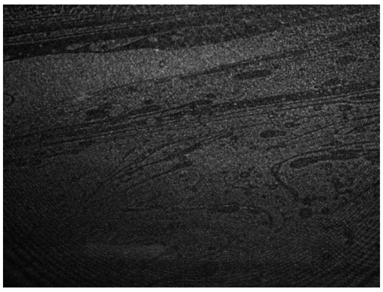<br>0.25 mNm^{-1}	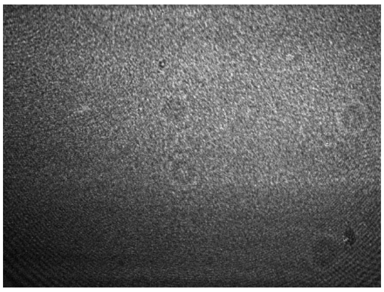<br>14.80 mNm^{-1}

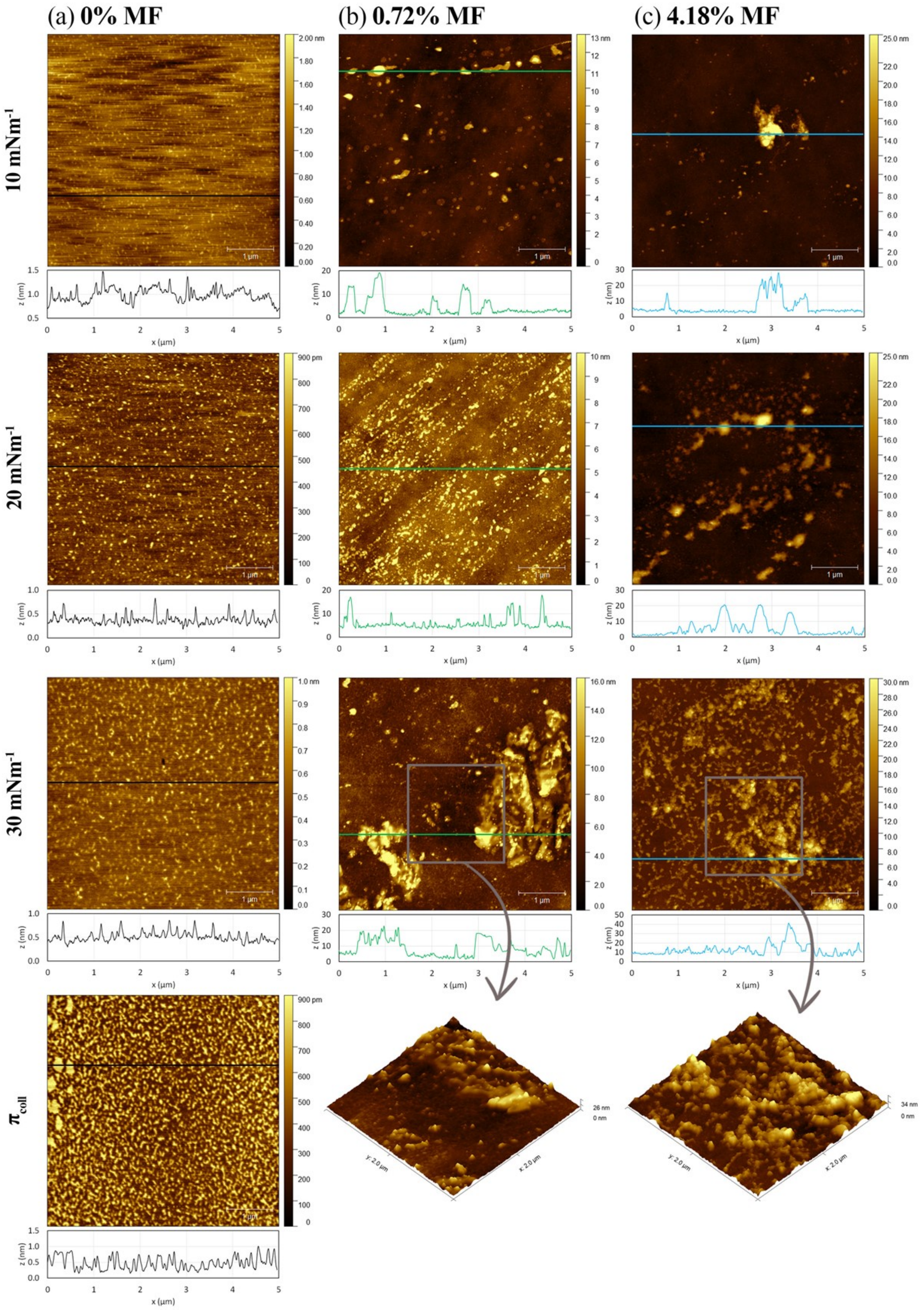

Figure S8: Effect of various concentrations of mometasone furoate on the molecular organization of mixed Langmuir-Blodgett films of DPPC:POPC:POPG:CHOL, 60:20:10:10 studied by the AFM micrographs and cross-sections. Column a presents data for the basic system transferred at characteristic surface pressures (π) of 10, 20, 30, and 38 mNm^{-1} (close to the collapse surface pressure). Columns b and c show AFM topographical images and cross-sections of the basic system mixed with 0.72 and 4.18% MF, respectively, at π of 10, 20, and 30 mNm^{-1}. 3-D images were added to visualize the characteristic regions of the samples. The lines in different colors indicate cross-section locations. The AFM scan area was 5 × 5 μm.

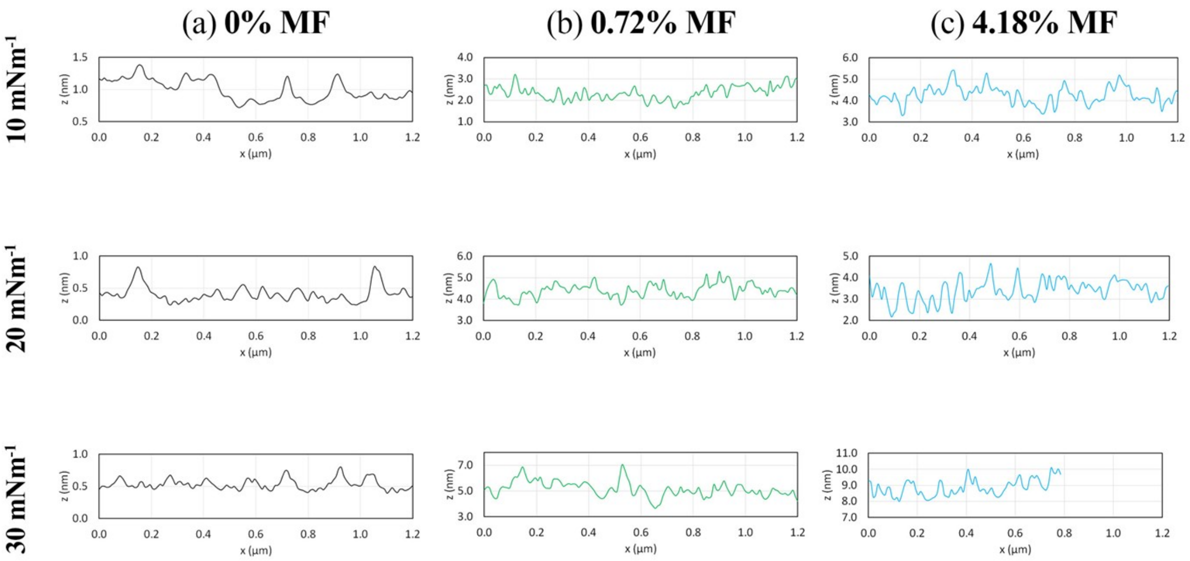

Figure S9: Effect of various concentrations of Mometasone Furoate on the profiles of the mixed Langmuir-Blodgett films of DPPC:POPC:POPG:CHOL 60:20:10:10 studied by AFM cross-sections. Column A. presents data for the basic system transferred at characteristic surface pressures (π) of 10, 20, 30, and 38 mNm^{-1} (close to the collapse surface pressure). Columns B and C represent systems with 0.72 and 4.18% MF, respectively, at π of 10, 20, and 30 mNm^{-1}. For samples containing the MF, cross-sections were located within aggregate-free areas.

S1.3 Lipid order parameter calculation

The order parameter is a measurement of the structural orientation of phospholipids in lipid monolayers. It is calculated by the following equation (Eq. 1s),

$$S_z = \frac{1}{2}\left(3\langle\cos^2\theta\rangle - 1\right) \tag{1s}$$

where S_z be the order parameter, θ be the angle between lipid tails and monolayer normal [15], angular brackets specify the mean of temporal and molecular ensembles [16]. The values of S_z ranging from -0.5 to 1.0 indicate that when $S_z = 1$, then the lipid tails are perfectly aligned with the monolayer normal (along the *z*-axis), whereas $S_z = -0.5$ implying the lipid tails are completely anti-align along monolayer normal. In our study, the Python script *do-order-gmx5.py* (MARTINI website) is used to determine order parameters [17]. The order parameter gives information about the phase behavior of the monolayer. The higher value of order parameter ($Sz = 1$) indicates that the monolayer is in a liquid condense (LC) phase representing the highly ordered lipids chains along the monolayer normal (*z-axis*) and unlike the lipids in a liquid expanded (LE) phase at lower value of order parameter.

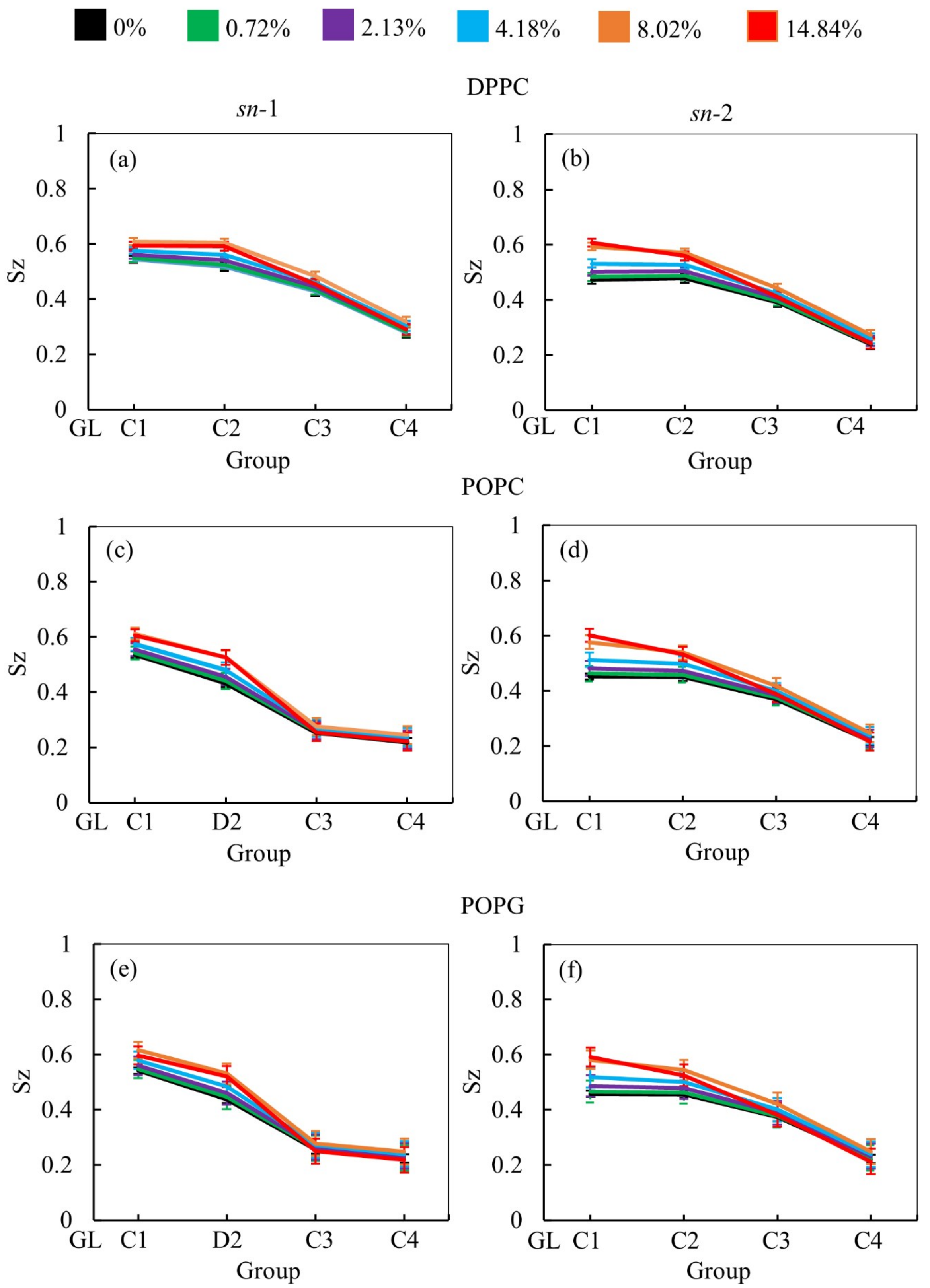

Figure S10: Order parameter of the lipids; DPPC (a, b), POPC (c, d), and POPG (e, f) for *sn*-1 and *sn*-2 chains in columns at increasing drug concentrations for the APL value 61 Å^2. The

values were calculated for the last 1-μs of the 2-μs simulations. The standard deviations were estimated using the frames of trajectories.

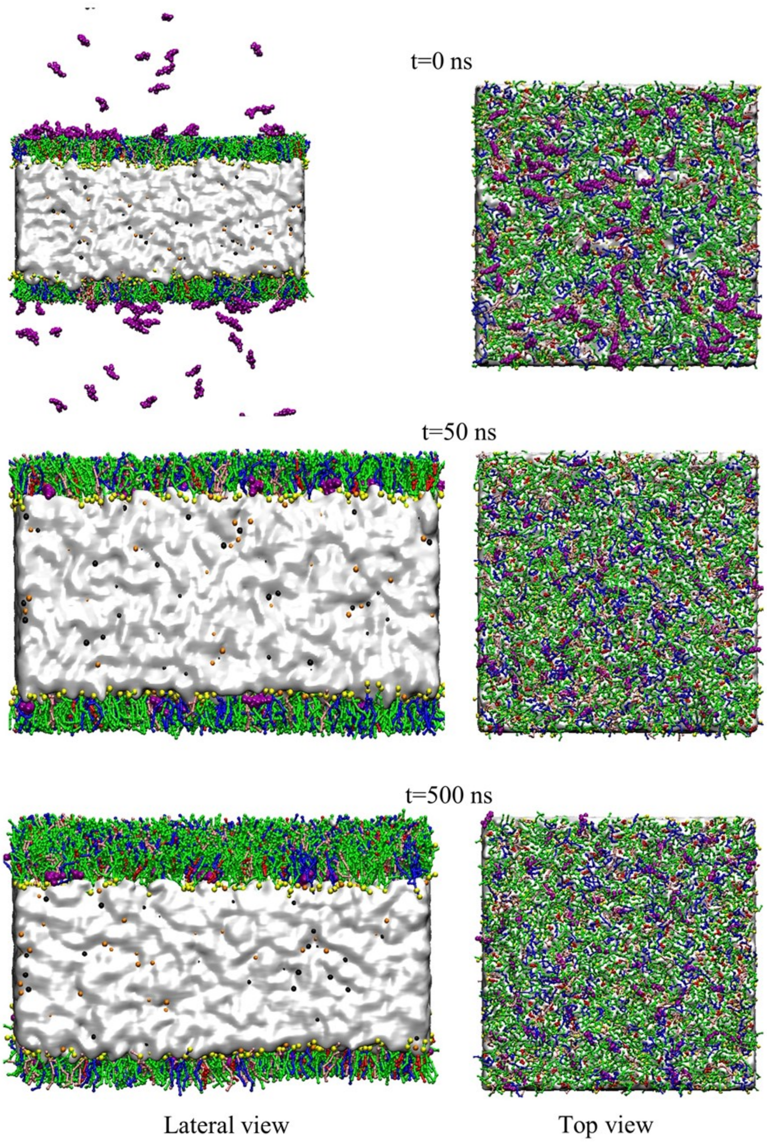

Figure S11: Surfactant and drug molecules distribution on the monolayer at APL value of 53 Å^2 for the drug concentration 4.18% w/w. The snapshots were captured at different simulation times from both the lateral and top view of the monolayer. DPPC is pointed by green, POPC in blue, POPG in pink, cholesterol in red, MF in purple, water in white, Na^+ in orange and Cl^- in black as well as lipids head group in yellow.

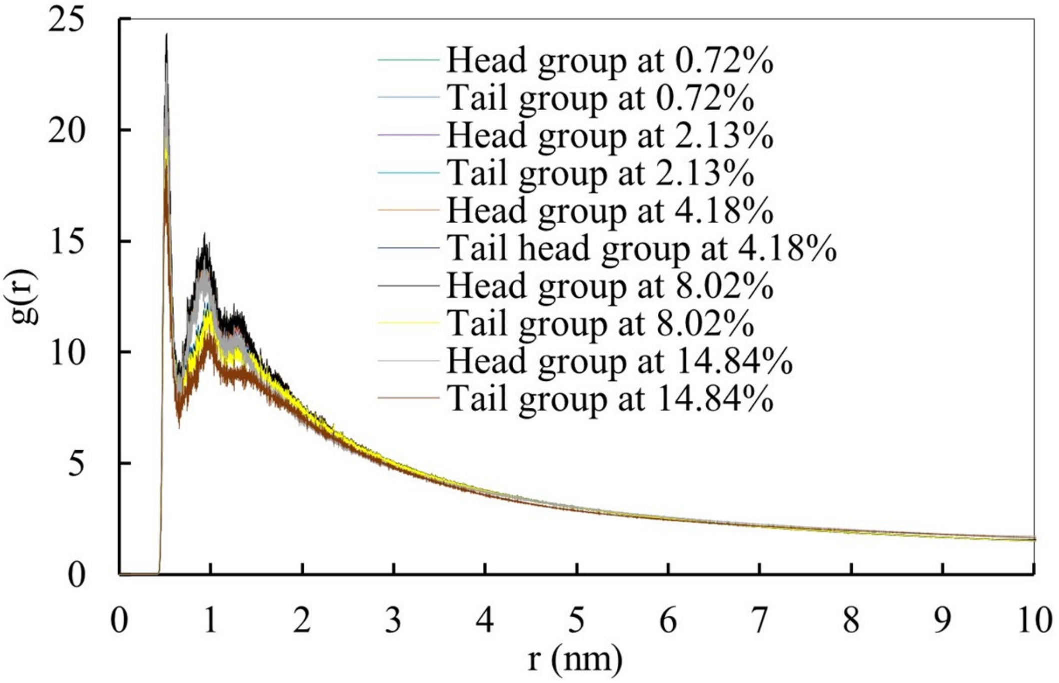

Figure S12: The radial distribution function (RDF) between drug molecules and lipid monolayer (lipid head and lipid tails) at different drug concentrations within a cutoff distance 1.0 nm during lung compression (APL value of 61 Å^2).

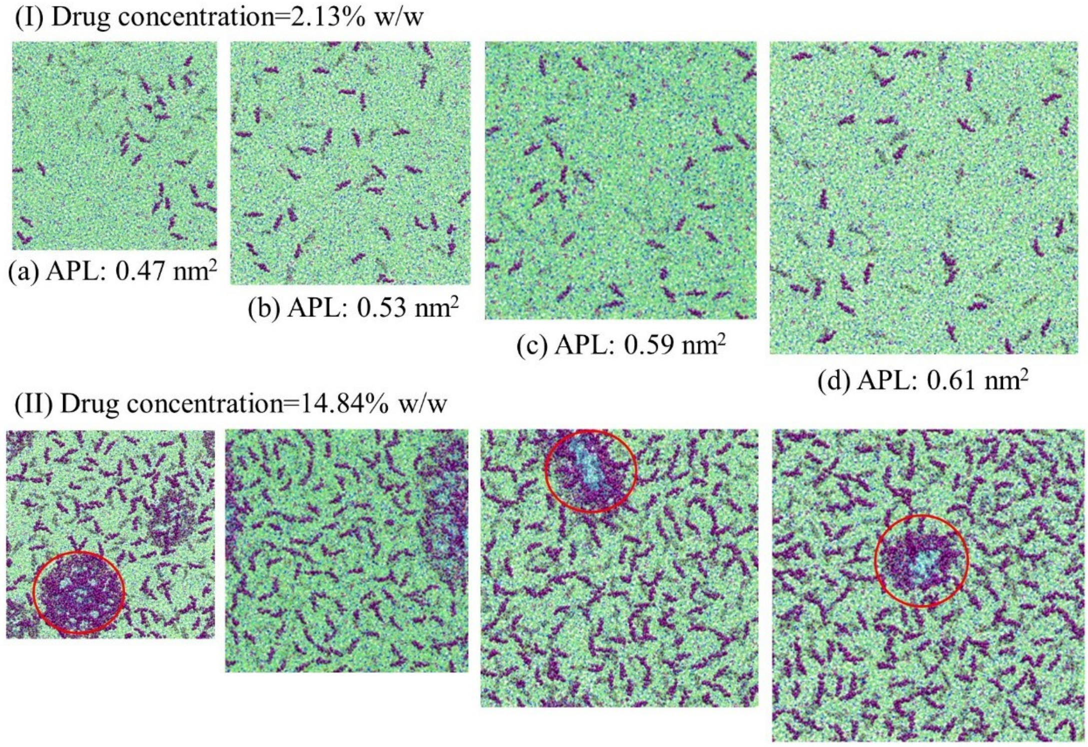

Figure S13. Snapshots of simulation system at (a) APL=0.47 nm^2 (highly compressed monolayer), (b) APL=0.53 nm^2 (intermediate state, i.e., compressed-expanded monolayer), (c) APL=0.59 nm^2 and (d) APL=0.61 nm^2 (expanded monolayer) in the presence of (I) 2.13% w/w and (II) 14.84% w/w drug concentrations. The drug molecules are shown in purple color.

References:

1. Ortiz-Collazos, S., et al., *Influence of levofloxacin and clarithromycin on the structure of DPPC monolayers.* Biochimica et Biophysica Acta (BBA)-Biomembranes, 2019. **1861**(10): p. 182994.
2. Hu, J., et al., *Investigation of Drug for Pulmonary Administration–Model Pulmonary Surfactant Monolayer Interactions Using Langmuir–Blodgett Monolayer and Molecular Dynamics Simulation: A Case Study of Ketoprofen.* Langmuir, 2019. **35**(41): p. 13452-13460.
3. Estrada-López, E.D., et al., *Prednisolone adsorption on lung surfactant models: insights on the formation of nano aggregates, monolayer collapse and prednisolone spreading.* RSC Advances, 2017. **7**(9): p. 5272-5281.
4. Islam, M.Z., et al., *Concentration-Dependent Effect of the Steroid Drug Prednisolone on a Lung Surfactant Monolayer.* Langmuir, 2022. **38**(14): p. 4188-4199.
5. Islam, M.Z., et al., *The steroid mometasone alters protein containing lung surfactant monolayers in a concentration-dependent manner.* Journal of Molecular Graphics and Modelling, 2022. **111**: p. 108084.
6. Stachowicz-Kuśnierz, A., et al., *The lung surfactant activity probed with molecular dynamics simulations.* Advances in Colloid and Interface Science, 2022. **304**: p. 102659.
7. Hossain, S.I., et al., *The role of SP-B1-25 peptides in lung surfactant monolayers exposed to gold nanoparticles.* Physical Chemistry Chemical Physics, 2020. **22**(27): p. 15231-15241.
8. Ortiz-Collazos, S., et al., *Interaction of levofloxacin with lung surfactant at the air-water interface.* Colloids and Surfaces B: Biointerfaces, 2017. **158**: p. 689-696.
9. Martini. *General Purpose Coarse-Grained Force Field.* [cited 2024; Available from: http://www.cgmartini.nl/index.php/tutorials-general-introduction-gmx5/parametrzining-new-molecule-gmx5.
10. Alessandri, R., et al., *Martini 3 Coarse-Grained Force Field: Small Molecules.* Advanced Theory and Simulations, 2022. **5**(1): p. 2100391.
11. Wishart, D. *DrugBank.* 2006 [cited 2024; Available from: https://go.drugbank.com/drugs/DB14512.
12. Wishart, D. *DrugBank.* 2006 [cited 2020; Available from: https://go.drugbank.com/drugs/DB00764.

13. Tetko, I.V., et al., *Virtual computational chemistry laboratory–design and description.* Journal of Computer-Aided Molecular Design, 2005. **19**(6): p. 453-463.
14. *Chem Axon. Chemicalize*. April 22, 2021]; Available from: https://chemicalize.com/welcome.
15. Hu, Q., et al., *Effects of graphene oxide nanosheets on the ultrastructure and biophysical properties of the pulmonary surfactant film.* Nanoscale, 2015. **7**(43): p. 18025-18029.
16. Vermeer, L.S., et al., *Acyl chain order parameter profiles in phospholipid bilayers: computation from molecular dynamics simulations and comparison with 2 H NMR experiments.* European Biophysics Journal, 2007. **36**(8): p. 919-931.
17. Monticelli, L., et al., *The MARTINI coarse-grained force field: extension to proteins.* Journal of Chemical Theory and Computation, 2008. **4**(5): p. 819-834.
